# HCLmNet: A unified hybrid continual learning strategy multimodal network for lung cancer survival prediction

**DOI:** 10.1371/journal.pone.0316509

**Published:** 2026-03-24

**Authors:** MD Ilias Bappi, David J. Richter, Shivani Sanjay Kolekar, Kyungbaek Kim

**Affiliations:** Department of Artificial Intelligence Convergence, Chonnam National University, Gwangju, South Korea; Khatam University, IRAN, ISLAMIC REPUBLIC OF

## Abstract

Lung cancer survival prediction remains one of the most challenging tasks in modern healthcare, as accurate and adaptive prediction models are essential for improving patient outcomes. However, the continuous inflow of new patient data in hospital environments demands models that can update incrementally without losing prior knowledge a challenge known as catastrophic forgetting. This problem is compounded by the complexity of multimodal data integration, which combines heterogeneous sources such as CT and PET imaging, genomic (DNA) sequences, and clinical records. Traditional deep learning (DL) models, especially CNN-based systems, often fail to capture subtle patterns such as ground-glass opacities or multi-lesion tumors and cannot effectively adapt to new data streams. To overcome these challenges, this study proposes HCLmNet, a Hybrid Continual Learning (CL) Multimodal Network that integrates Elastic Weight Consolidation (EWC) with three complementary replay-based modules: Experience Replay (ER), Instance-Level Correlation Replay (EICR), and Class-Level Correlation Replay (ECCR). ER stabilizes learning through selective sample replay; EICR preserves fine-grained inter-instance relationships across modalities; and ECCR employs triplet-based contrastive learning to maintain class-level correlations. The architecture incorporates a Swin Transformer (SwinT) for extracting critical imaging features, XLNet for modeling DNA patterns, and a Fully Connected Network (FCN) for processing temporal clinical data. A cross-attention fusion layer integrates these modalities, while an FCN and Cox Proportional Hazards (CoxPH) model produce final 5-year survival predictions. Experimental results on multimodal lung cancer datasets show that traditional models such as CoxPH and DeepSurv achieved Concordance Index (C-index) scores of 0.65 and 0.70, respectively. The base multimodal model without CL achieves a C-index of 0.76 and a Mean Absolute Error (MAE) of 189 days. In contrast, the proposed HCLmNet, equipped with CL mechanisms, reaches a C-index of 0.84, representing a 7.7% improvement over the best baseline. Furthermore, the model reduces the MAE from 252 and 189 days to 140 days and minimizes catastrophic forgetting to 0.08. These improvements stem from the synergistic integration of the ER, EICR, and ECCR CL modules, which enable the model to retain prior knowledge while effectively adapting to new data. Overall, HCLmNet demonstrates superior stability, adaptability, and interpretability for lung cancer survival prediction in dynamic clinical environments.

## 1 Introduction

Lung cancer is one of the most common malignancies worldwide and accounts for approximately 18% of cancer-related deaths. [1] Early detection, accurate monitoring, and effective treatment planning all rely on comprehensive medical data such as clinical patient information, computed tomography (CT), positron emission tomography (PET), and genomic mutation data including single nucleotide variants (SNV), heterozygous (HETE), and homozygous (HOMO) alterations. Traditionally, radiologists and medical experts perform visual inspections of imaging studies alongside patient history and experimental records [2,3]. However, this process is time-intensive and subject to human error: survival time predictions and treatment decisions frequently depend on clinicians’ subjective experience and may lack consistency or reproducibility.

While DL models have shown promise in improving predictive accuracy, they encounter notable challenges: convolutional neural networks (CNNs) often struggle to detect small or multiple tumour instances in high resolution medical images [4,5], and integrating heterogeneous data sources clinical, imaging, and genomic remains difficult because of disparities in feature representation and modality-specific characteristics [6]. An equally critical challenge in survival prediction pertains to the use of CL, where models must integrate new patient data over time without overwriting prior knowledge a phenomenon known as catastrophic forgetting [7]. CL methods have recently emerged as a viable solution for evolving datasets [8]. Broadly speaking, existing CL strategies fall into three categories: regularization-based methods [9,10], structure-based approaches [11–14], and replay-based techniques [15–17]. Recent advances in multimodal learning have demonstrated that combining imaging, genomic, and clinical features substantially improves predictive accuracy compared to unimodal models [18–20]. However, most of these models are trained in static environments, limiting their ability to adapt to the dynamic nature of clinical data, where new patients, imaging protocols, or modalities are continuously introduced. In such evolving hospital settings, retraining models from scratch is computationally expensive and inefficient. Moreover, when data volumes grow significantly, relying solely on replay buffers without effective regularization or parameter-protection mechanisms often results in performance degradation and instability during incremental updates. In particular, replay-based methods mimic human learning by selectively storing and rehearsing past samples, offering a computationally efficient means of alleviating catastrophic forgetting. However, conventional replay systems often overlook critical correlations at both the instance and class levels, which are essential for maintaining structural consistency across tasks. Moreover, when new datasets are large or complex, relying solely on a memory buffer becomes impractical; its size and efficiency deteriorate, leading to performance degradation. In such scenarios, the complementary integration of Elastic Weight Consolidation (EWC) provides an effective solution by regularizing parameter updates and protecting knowledge learned from previous tasks.

In this context, continual learning has emerged as a crucial paradigm for developing adaptive and resilient medical AI systems [21]. CL enables models to incrementally learn from new patient data while retaining previously acquired knowledge, thereby ensuring longitudinal stability and interpretability, both vital for clinical deployment. Despite its promise, most existing CL methods in healthcare remain limited by shallow replay strategies, insufficient modeling of inter-instance and inter-class correlations, and poor scalability when handling high-dimensional multimodal datasets. These limitations highlight the need for a robust hybrid CL framework capable of sustaining performance over time and effectively integrating heterogeneous modalities such as CT, PET, clinical records, and genomic data. To fill these gaps, this work suggests a hybrid CL framework that integrates EWC with three replay–phase modules: ER, EICR, and ECCR. ER serves to maintain foundational knowledge by replaying mixtures of historical and new tasks; EICR preserves refined inter-instance relationships through a correlation matrix, safeguarding structural consistency; and ECCR ensures class-level boundaries are maintained via contrastive learning and random triplet mechanisms (anchor, positive, negative), thus preserving distinctions such as tumour vs. non-tumour regions across tasks. This hybrid CL strategy is embedded within a multimodal architecture: clinical data are managed via an FCN network, imaging features (CT, PET) are captured using a SwinT to address the limitations of CNNs in detecting fine tumour features, and genomic data are processed via an XLNet-permutation model to leverage small DNA datasets and latent patterns. A novel cross-attention fusion layer integrates clinical, imaging, and genomic embeddings, which then flow into a FCN and are finalized via a CoxPH model for survival prediction (see Fig 2). In summary, the key contributions are as follows:

- A hybrid CL framework is introduced, integrating EWC with replay-phase mechanisms to enable dynamic adaptation and effective retention of prior knowledge in lung cancer survival prediction.
- Three replay and EWC-based modules ER, EICR, and ECCR are developed within the framework to preserve foundational knowledge, inter-instance relationships, and class-level consistency, thereby enhancing scalability to large or complex multimodal datasets.
- A SwinT-based feature extraction strategy is employed to improve the detection of critical lung cancer characteristics, including ground-glass opacities and small tumour instances in CT and PET images.
- An XLNet permutation-based approach is leveraged to process limited DNA mutation datasets effectively, uncover latent genomic patterns, and enrich multimodal survival prediction.
- Inspired by cross-attention fusion techniques, clinical, CT, PET, and DNA modalities are integrated into a unified embedding representation, enabling comprehensive survival prediction through CoxPH modelling and adaptive CL techniques.
- Extensive ablation and comparative analyses with SOTA models are conducted, demonstrating that the hybrid CL-based multimodal framework provides superior performance and robustness.

The rest of this paper is organized as follows: Section 2 reviews related work on survival prediction, multimodal fusion, and CL. Section 3 outlines the theoretical background and clinical motivation underlying this study. Section 4 describes the proposed HCLmNet framework and its methodological components in detail. The experimental setup, datasets, and evaluation metrics are presented in Section 5. Section 6 reports the experimental results and analysis, including ablation studies, comparative evaluation, and validation of the proposed model. Section 7 provides an in-depth discussion, covering their clinical interpretation, comparison with prior work, and identified limitations. Finally, Section 8 concludes the paper and outlines directions for future research.

## 2 Related work

### 2.1 Survival prediction models

Survival prediction in lung cancer is a critical component of personalized treatment planning and outcome monitoring. Early studies used clinical and imaging data to estimate short-term survival: for example, Sesen et al. [22] used the LUCADA dataset to predict 1-year survival and recommend treatments, while Yu et al. [23] modelled survival distributions across dependent tasks. Paul et al. [24] demonstrated how CNN-extracted CT features can be used with nearest-neighbor classifiers for survival estimation. More recently, DL approaches such as DeepSurv [25] and other neural Cox-based frameworks [26] have improved performance by capturing nonlinear relationships in large-scale data. However, these models typically assume static datasets and often overlook evolving patient populations and continuous data influx. Recent studies have emphasized the growing role of imaging and multimodal learning in survival estimation. Meng et al. [27] proposed an adaptive segmentation-to-survival framework (AdaMSS) for PET/CT imaging, learning modality-specific fusion strategies and achieving C-indices exceeding 0.80 on multi-center cohorts. Li et al. [28] introduced a pan-cancer multimodal fusion model that integrates imaging, clinical, and omics data through cross-attention, demonstrating high generalizability across tumor types [29]. In this context, the 5-year survival period is adopted as the prediction standard, following the convention established by the American Cancer Society (ACS) and the Surveillance, Epidemiology, and End Results (SEER) Program. The 5-year survival rate serves as a widely accepted benchmark for evaluating patient prognosis, comparing treatment effectiveness, and assessing long-term outcomes in oncology research and clinical practice [30,31]. Importantly, imaging modalities such as CT and PET contribute unique prognostic value by capturing anatomical tumor volume, metabolic activity (SUV uptake), and spatial heterogeneity, providing crucial information beyond clinical and genomic features for long-term survival modeling.

### 2.2 Multimodal fusion strategies

The integration of heterogeneous data modalities, clinical records, imaging (CT/PET), and genomics has demonstrated strong potential in improving survival prediction accuracy. For example, Zhang et al. [32] introduced a multimodal transformer architecture combining histopathology and genomic features for cancer prognosis. Farooq et al. [6] extended this direction in lung cancer by fusing CT imaging, mutation profiles, and clinical metadata. Cross-modal attention mechanisms are increasingly adopted for instance, Yang et al. [33] proposed a transformer-based scheme to dynamically weight modality contributions, achieving significant performance gains. More recently, Simon et al. [20] reviewed the future of multimodal AI in radiology, highlighting graph neural networks and transformers as dominant architectures for integrating clinical and imaging data. Similarly, Wu et al. [34] proposed Lite-ProSENet, a cross-modality network for NSCLC prognosis that learns adaptive modality dependencies instead of fixed fusion heuristics [35]. Imaging modalities (CT and PET) are central to these frameworks: CT provides high-resolution anatomical and morphological context, while PET captures functional and metabolic activity, allowing a richer representation of tumor burden and biological behavior [36]. Yet, despite these advances, most fusion models remain trained offline in batch settings and fail to adapt to incremental data updates or domain shifts over time.

### 2.3 Continual learning frameworks

Continual Learning is essential in clinical predictive systems where new patient cohorts, imaging protocols, or modalities may arrive over time. CL methods aim to learn from streaming data while preserving prior knowledge, mitigating the phenomenon of catastrophic forgetting [7]. The dominant CL paradigms include: (i) regularization-based methods like Elastic Weight Consolidation (EWC) [37], which penalize changes to critical parameters through the Fisher Information Matrix (FIM) that estimates parameter importance [38]; (ii) structure-based methods such as Progressive Neural Networks [11], which expand model capacity for new tasks; and (iii) replay-based methods like experience replay [16], which store and revisit prior samples. Recent surveys such as Kumari et al. [39] and Bruno et al. [19] provide systematic reviews of CL applications in medicine, emphasizing the need for multimodal and explainable CL frameworks. While CL has recently been explored in medical imaging [21], most works remain unimodal, focusing on classification rather than prognostic tasks and neglecting inter-instance or inter-class relational structures. Furthermore, model transparency and deployment security have emerged as critical issues. For example, Khan et al. [40] proposed a blockchain-integrated explainable AI (XAI) framework for ethical, personalized healthcare decision making is highlighting the intersection of CL, trust, and real-world deployment.

### 2.4 Summary of gaps and contribution

While survival models, multimodal fusion strategies, and CL techniques have each advanced the SOTA, none so far provide a unified framework that (i) combines deep multimodal inputs (clinical, imaging (CT/PE), and genomic), (ii) applies progressive learning over time, and (iii) ensures retention of previously learned knowledge without catastrophic forgetting. Most existing frameworks either lack temporal adaptability or disregard the evolving data landscape characteristic of real-world oncology. To fill these gaps, a hybrid CL framework is proposed that integrates a fixed-capacity memory buffer with ER, EICR, and ECCR modules under an EWC-based regularization scheme driven by the FIM. The offered model unifies robust feature extraction (via SwinT for imaging, XLNet for clinical data, and FCN for fusion), a cross-attention–based fusion layer to align multimodal representations, and a CL mechanism that enables dynamic patient updates. CT and PET imaging provide complementary anatomical and metabolic insights, which, when fused with clinical and genomic features, yield richer patient representations for accurate 5-year survival prediction through CoxPH [30,31].

## 3 Background

Since lung cancer is one of the leading causes of cancer related deaths worldwide, early and precise survival projections are crucial for enhancing patient outcomes and clinical judgment. These forecasts are essential for directing treatment decisions and improving care plans. A crucial component of this process is the TNM staging system, developed by the American Joint Committee on Cancer (AJCC) and the International Union Against Cancer (UICC) [41]. This globally recognized system assesses the severity and spread of cancer in the body, where T describes the size of the tumor, N represents the spread to nearby lymph nodes, and M indicates metastasis to other body parts. In addition to TNM staging, other patient-specific attributes such as gender, age, smoking status and amount, survival time, and overall clinical stage play a critical role in survival prediction. Together, these multimodal data points offer a comprehensive view of a patient’s condition, enabling clinicians to make informed decisions about treatment strategies. However, leveraging these diverse data types to predict patient survival poses significant challenges in dynamic hospital environments. New patient data, including imaging, DNA, and updated medical records, is generated daily, requiring predictive models to adapt continually without losing prior knowledge. Conventional models for DL, while effective in processing complex datasets, often struggle with catastrophic forgetting where learning from new data overwrites previously acquired knowledge. This limitation undermines the reliability of survival predictions and hampers the integration of multimodal data in rapidly evolving clinical settings. To address this, hybrid CL strategies have emerged as a promising solution. Replay-based methods, which use memory buffers to retain critical information from previous data, are particularly effective for incremental learning. However, replay-based approaches alone may not be sufficient when the new data is big or complicated, such as when imaging, DNA profiles, and behavioral records are combined to predict lung cancer. [42]. In these situations, EWC offers a complementary approach, preserving important parameters from previous tasks by penalizing updates to critical weights. When integrated with replay-phase strategies, EWC enhances the model’s ability to adapt to new data while maintaining prior knowledge, ensuring more robust and reliable performance. For instance, imagine a hospital managing the care of a lung cancer patient. Integrating TNM stages, imaging data, and behavioral attributes such as smoking history into a predictive model could help clinicians project the patient’s five-year survival probability, enabling timely interventions and tailored care. Without robust hybrid CL strategies, the model’s predictions might falter as it struggles to balance new and existing knowledge. By combining replay-based methods with EWC, these challenges can be mitigated, advancing the field of survival prediction and ensuring predictive models remain reliable, adaptable, and effective in modern healthcare environments.

**Fig 1:**
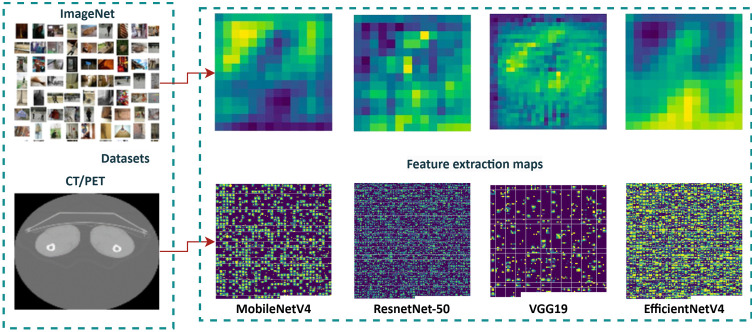
Comparison of feature extraction performance across CNN models (MobileNetV4, ResNet-50, VGG19, and EfficientNetV4) trained on ImageNet versus a medical imaging dataset. The visualization maps highlight the differences in feature focus, with ImageNet-trained models demonstrating limited sensitivity to fine-grained details.

Another challenge is accurate feature extraction from medical imaging is critical for lung cancer survival prediction, as CT and PET scans provide valuable insights into tumor size, texture, and spread. Although, traditional methods, particularly those relying on CNNs, face significant limitations. CNNs are quite good at seeing patterns in pictures, but they frequently have trouble capturing microscopic or subtle features like tiny cancers or ground-glass opacities, which are crucial in lung cancer diagnosis and prognosis. The challenge arises because these models are typically pretrained on datasets like ImageNet, which do not capture the complex, specialized features found in medical imaging. This makes them less effective for medical applications, where the data has unique characteristics that require tailored models. Additionally, CNNs tend to focus primarily on local features, resulting in the loss of adjacent pixel and vertex information when images are resized or processed. This can hinder the detection of critical tumor features embedded in the broader anatomical context. For instance, as illustrated in Fig 1, a comparison of CNN models such as MobileNetV4, ResNet-50, VGG19, and EfficientNetV4 reveals suboptimal performance in capturing fine-grained medical imaging features between ImageNet. Feature visualization maps for these models demonstrate their dominant focus on high-level patterns rather than the intricate details needed for precise lung cancer analysis.

To overcome these limitations, advanced techniques like the SwinT offer a promising alternative. Unlike Vision Transformers (ViT), which employ global attention across the entire image, the SwinT introduces Shifted Window Attention to partition images into non-overlapping windows and apply self-attention locally within these regions. This localized attention mechanism allows the SwinT to efficiently capture fine details, such as tiny tumors, while also preserving the ability to learn long-range dependencies across the image. This architectural advantage is particularly relevant for medical imaging tasks, as it enables the detection of small tumor features within a larger anatomical structure critical for accurate staging and treatment planning in lung cancer. Furthermore, the SwinT’s ability to adapt to diverse medical imaging datasets ensures that models are not limited by biases inherent in pretrained datasets like ImageNet. By incorporating such cutting-edge techniques, this research aims to enhance imaging feature extraction and ultimately improve lung cancer survival prediction in real-world clinical settings including CL strategies.

## 4 Method

**Fig 2:**
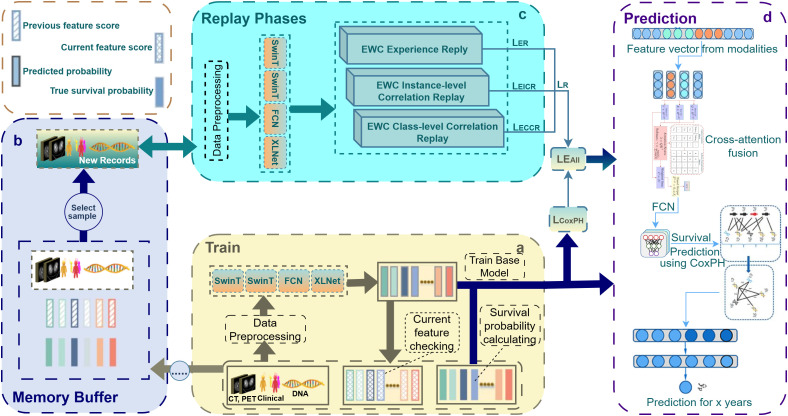
Overview of the proposed HCLmNet framework. The model operates through four phases: **(a) Training**, where CT, PET, clinical, and DNA data are preprocessed and encoded using SwinT, FCN, and XLNet; **(b) Memory Buffer**, which stores representative samples from previous tasks; **(c) Replay**, where ER, Instance-level (EICR), and Class-level(ECCR) mitigate catastrophic forgetting through hybrid EWC-based constraints; and **(d) Prediction**, where fused multimodal features pass through a cross-attention fusion module and a CoxPH layer for survival probability estimation. A legend summarizing feature states and probability terms is provided in the upper right corner for visual reference.

This study introduces a novel hybrid CL strategy that combines EWC and Replay-based methods within a multimodal network, enabling incremental updates of model parameters and refinement of survival predictions for lung cancer patients. The framework integrates both previously trained knowledge and newly acquired CT, PET, clinical, and DNA data. The approach employs a SwinT-based feature extraction mechanism that overcomes limitations of ImageNet-pretrained CNN models, which often fail to capture critical features such as ground-glass details and multiple tumor instances in CT and PET scans. Additionally, permutation-based XLNet techniques are leveraged to learn contrastive patterns in DNA data, mitigating the challenges posed by the limited size of DNA datasets within large-scale multimodal settings. Clinical data are processed through an FCN network for sequential learning. To achieve adaptive and accurate survival prediction, clinical, CT, PET, and DNA data are integrated through a cross-attention fusion mechanism, complemented by an FCN for CoxPH modeling and supported by a robust CL framework. The methodological overview of the proposed framework is illustrated in Fig 2.

### 4.1 Hybrid incremental learning process

The process begins by training the base model using clinical, CT, PET, and DNA data. Clinical and imaging data are processed through FCN, SwinT, and XLNet, with a cross-attention mechanism integrating all modalities into the CoxPH framework for seamless prediction. During this phase, a replay memory (RM) buffer is initialized to store previously learned data, ensuring that valuable knowledge is preserved. As new data becomes available, it is preprocessed and used to update the memory buffer, where both old data (from previous training) and new data (from the most recent batch) are stored. The model retrains incrementally using data from the memory buffer, which includes both old and new data, to prevent catastrophic forgetting. The loss function during incremental training consists of two key components: the standard loss, which is based on the new data, and the EWC loss, which penalizes significant changes in the model’s weights to preserve previously learned knowledge. The total loss is calculated as:


$${ℒ}_{\text{total}}={ℒ}_{\text{new}}+\lambda \sum_{i}F_{i}(\theta_{i}-\theta_{i}^{*}{)}^{2}$$ (1)

Where:

- ${ℒ}_{\text{new}}$ is the standard loss for the new data.
- λ is a hyperparameter controlling the strength of the EWC penalty.
- *F*_*i*_ is the Fisher information for the *i*-th model parameter, which reflects the importance of that parameter in retaining knowledge.
- $\theta_{i}$ is the current value of the parameter, and $\theta_{i}^{*}$ is the value from the original model.

**Fig 3:**
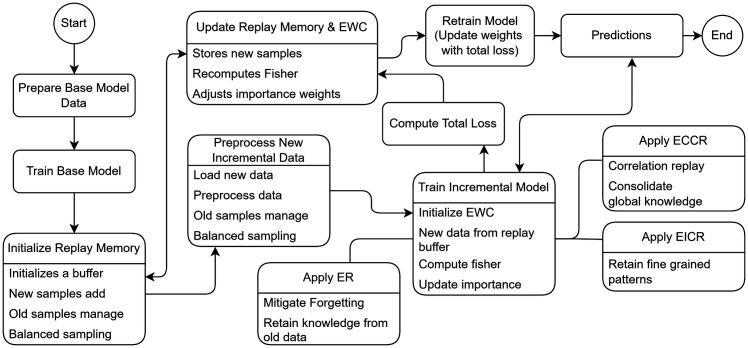
Overview of the Hybrid continual learning flowchart for incremental survival prediction.

In addition to EWC, techniques such as ER, EICR, and ECCR are integrated into the training process. The ER module aids in replaying representative samples from the memory buffer, mitigating the interference from new data by ensuring that important past information is not lost. The EICR module preserves fine-grained feature patterns by constructing a correlation matrix that captures inter-instance relationships, helping maintain structural information critical to individual data points. The ECCR module consolidates global knowledge by preserving inter-class relationships, achieved through balanced sampling and a triplet loss function that ensures clear boundaries within classes. FIM plays a crucial role in EWC by determining the significance of each parameter’s adjustment. It does this by calculating the second-order derivatives of the loss function with respect to each parameter, guiding the penalty for weight changes during incremental learning. This hybrid CL framework ensures that the model adapts to new data while retaining knowledge from previous tasks. At the conclusion of this process, the CoxPH model is used to predict the 5-year survival probability for each patient. This process is depicted in Fig 3, and a detailed explanation of each hybrid technique is provided in Fig 7.

### 4.2 Clinical modality processing with FCN

16 clinical features were utilized, including demographic, clinical staging, and behavioral factors, for survival prediction. These features were preprocessed using standard tabular techniques. In this process, the FCN is designed to generate a token embedding (token_dim = 64) that aligns with the embeddings of other modalities. The 16 clinical features (input_dim = 16) pass through the FCN, which progressively reduces dimensionality across layers with 512, 256, 128, and finally 64 neurons. Each layer incorporates batch normalization for stability, ReLU for non-linearity, and Dropout (0.3) to minimize overfitting. This hierarchical structure captures increasingly abstract features, ensuring efficient feature representation. The final 64-dimensional token was chosen empirically to balance computational efficiency and performance. Its compatibility with other modalities, which also output 64-dimensional tokens, ensures seamless integration during multimodal fusion. Mathematically, given the input $X\in {\mathbb{R}}^{N\times D}$, where *N* is the batch size (32 samples per batch) and *D* = 16 is the feature dimension, the FCN reduces *D* through hierarchical transformations as follows [43]:


$$f_{\text{out}}=W_{k}\cdot \sigma \left(W_{k-1}\cdot \sigma \left(\ldots W_{1}\cdot X+b_{1}\right)+b_{k-1}\right)+b_{k}$$ (2)

Where: $W_{i},b_{i}$ represent the weights and biases of the *i* -th layer, σ is the ReLU activation function, $f_{\text{out}}\in {\mathbb{R}}^{N\times 64}$ is the final token embedding.

These embedding tokens effectively represent the clinical features while maintaining consistency with the token outputs from other modalities. These tokenized clinical features are subsequently passed to the cross-attention fusion block, where they are integrated with the tokenized outputs from CT, PET, and DNA data. This cross-modality integration enhances the survival prediction model by combining temporal insights from the clinical data with spatial features from imaging modalities and genomic data, ultimately improving the survival prediction process. The stage of FCN in the framework is illustrated in Fig 2.

### 4.3 DNA modality processing with XLNet

In the multimodal framework, XLNet is employed to process DNA features such as counts of SNVs, HOMO, and HETE variants to extract meaningful patterns relevant to lung cancer prediction. To support this objective, XLNet is configured with parameters designed to capture complex dependencies within genomic data. The vocabulary size is defined based on the number of unique tokens present in the dataset. The model consists of 6 layers, each containing 4 attention heads. Each attention head has a dimensionality of 8, while the inner feed-forward layer dimension is set to 32, enabling the extraction of intricate relationships among DNA features. The overall model dimensionality is fixed at 32, determining the embedding size for each token. Permutation-based autoregressive modeling is adopted, in which each token *x*_*t*_ is predicted conditioned on preceding tokens within a randomly permuted sequence, allowing the model to learn robust and order-agnostic genomic patterns. The task is formulated as:


$$p_{\theta }(x_{z_{t}}|x_{z<t})$$ (3)

Here, $p_{\theta }$ denotes the probability of predicting the token $x_{z_{t}}$ at position *z*_*t*_, given the preceding tokens *x*_*z* < *t*_. This permutation-based approach allows XLNet to model bidirectional context efficiently by learning from all possible orderings of the sequence. By leveraging a multi-head attention mechanism to capture different aspects of relationships between tokens at various positions. The attention mechanism in each layer represented as:


$$\text{Attention}(Q,K,V)=\text{softmax}\left(\frac{QK^{T}}{\sqrt{d_{k}}}\right)V$$ (4)

where *Q* is the query matrix, *K* is the key matrix, *V* is the value matrix, and *d*_*k*_ is the dimension of the key vectors. In this configuration, 4 attention heads are employed, with each head having a dimensionality of 8, enabling the model to capture diverse relationships among features such as SNVs, HOMO, and HETE variants. The multi-head mechanism enables the model to learn complex dependencies at multiple levels of granularity. Through the self-attention mechanism, it learns latent patterns in the DNA data by attending to different parts of the input sequence. The self-attention layer updates the input embeddings by calculating:


$$g_{z_{t}}^{\left(m\right)}\leftarrow \text{Attention}(Q=g_{z_{t}}^{\left(m-1\right)},KV=h_{z<t}^{\left(m-1\right)};\theta )$$ (5)

$$h_{z_{t}}^{\left(m\right)}\leftarrow \text{Attention}(Q=h_{z_{t}}^{\left(m-1\right)},KV=h_{z<t}^{\left(m-1\right)};\theta )$$ (6)

**Fig 4:**
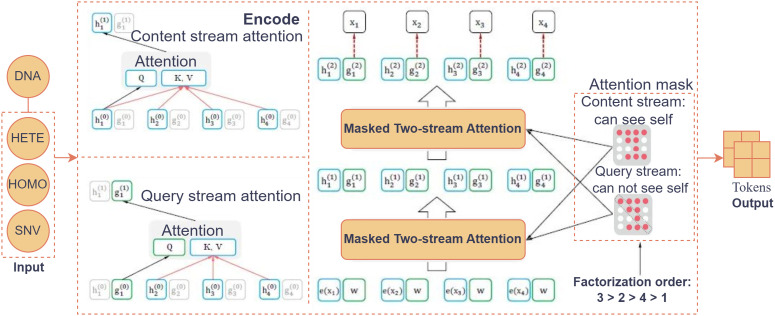
XLNet architecture for DNA data. The model processes SNV, HOMO, and HETE inputs by tokenizing the sequence and applying permutation-based autoregressive training to capture feature dependencies. A Two-Stream Self-Attention mechanism (Query Stream for prediction and Content Stream for context encoding) generates contextual token representations, which are later fused with other modality embeddings for survival prediction.

An iterative update process is utilized for both the query stream and the content stream, progressively refining the embeddings at each layer. The learned latent patterns are crucial for understanding the relationships between different genomic features. After processing the DNA data through multiple XLNet layers, the final output embeddings $h_{z_{t}}^{\left(M\right)}$ are projected into a 64-dimensional space through a linear transformation. This transformation is performed by a fully connected layer, as presented:


$$h_{z_{t}}^{\left(M\right)}\to Wh_{z_{t}}^{\left(M\right)}+b$$ (7)

where the output embeddings are multiplied by a learned weight matrix $W\in {\mathbb{R}}^{d_{\text{model}}\times 64}$, followed by an optional bias term $b\in {\mathbb{R}}^{64}$. This ensures that the final embedding dimension matches the required token size of 64. The final tokens are passed into the cross-attention fusion block, where they are merged with embeddings from other modalities, such as CT/PET and clinical, for multimodal integration, as shown in Fig 4. This fusion allows the model to leverage complementary information across modalities to improve prediction accuracy.

### 4.4 CT and PET modality processing with SwinT

The SwinT model is implemented as an efficient approach for processing high-resolution medical images, such as CT and PET scans. It addresses the limitations of ViT, particularly in terms of computational complexity, while offering improved performance in classifying fine-grained object details compared to traditional CNNs. This makes SwinT particularly well-suited for medical imaging tasks, where high accuracy in identifying complex structures, such as ground objects and tumors in CT/PET scans, is crucial [44]. The preprocessing pipeline for CT/PET images starts with an input shape of [1, 160, 128, 128], where:1 represents the batch size, 160 is the depth (number of slices), and 128×128 are the spatial dimensions of each slice. The patch embedding of The image is divided into non-overlapping patches of 4×4 pixels, resulting in tokens of shape [*N*, *C*], where $N=160\times 128\times 128/4^{2}=4096$ patches and *C* is the initial feature dimension. Each patch is flattened and projected to a higher-dimensional space via a linear embedding layer. In the Window Multi-Head Self-Attention (W-MSA) operation, attention is computed within fixed windows, thereby reducing computational complexity. Specifically, attention is calculated within windows of size M×M, which reduces the overall complexity to a linear relationship with respect to the number of patches:


$$O_{\text{W-MSA}}=2M^{2}hwC$$ (8)

After each W-MSA block, in Shifted Window Multi-Head Self-Attention (SW-MSA) windows are shifted to capture cross-window dependencies. This introduces quadratic complexity:


$$O_{\text{SW-MSA}}=4hwC^{2}$$ (9)

These steps enhance both local and global context modeling. After processing through several SwinT blocks, the output tokens are passed through a Multi-Layer Perceptron (MLP) to refine features, followed by Layer Normalization for dimensionality reduction. Finally, token embeddings $h_{z_{t}}^{\left(M\right)}$ are projected into a 64-dimensional space using a fully connected layer. This is performed by multiplying the embeddings with a learned weight matrix $W\in {\mathbb{R}}^{d_{\text{model}}\times 64}$, where *d*_model_ is the feature dimension before projection, followed by an optional bias term $b\in {\mathbb{R}}^{64}$. This ensures the output token size is reduced to 64, making it compatible for integration into downstream tasks like survival prediction:


$$h_{z_{t}}^{\left(M\right)}\times W+b$$ (10)

**Fig 5:**
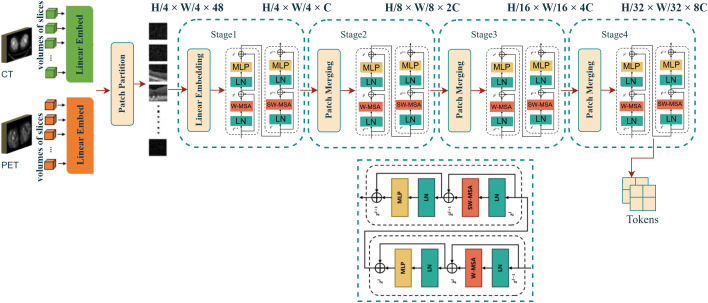
Overall architecture of the SwinT adapted for CT and PET scan analysis. The model uses two successive SwinT blocks that process CT and PET image patches to extract multi-scale feature representations. Its hierarchical design reduces computational complexity for high-resolution medical images, while the shifted-window self-attention mechanism captures long-range dependencies and fine-grained details across DICOM slices.

After tokenization and projection, the features from both CT and PET modalities are passed into a cross-attention fusion block, which combines the features with clinical, imaging, and mutation data. where the features from both modalities are merged using attention mechanisms. This fusion ensures that the combined representations are more informative and relevant for the downstream survival prediction task. The methodological overview of this process is illustrated in Fig 5.

### 4.5 Cross-attention fusion block

In the survival prediction framework, inspired by recent works such as [45], the cross-attention block is employed to integrate clinical, DNA, CT, and PET data by capturing dependencies both within and across these modalities. The data from each modality is first formatted into a consistent L×D structure, where *L* is the number of tokens or features, and *D* represents the feature dimension. Specifically, the CT and PET images are split into patches, tokenized, and flattened, resulting in a sequence of tokens (of length *L*_img_), where each token represents a patch encoded into *D* -dimensional space. For clinical and DNA tabular data, individual attributes or features are embedded directly into $L_{\text{tab}}\times D$, ensuring that all modalities share a common format, making cross-attention feasible.

The core of the cross-attention mechanism utilizes this unified structure to link information across modalities. The non-local operation within the cross-attention block is defined as:


$$\lambda_{kr}=g(u_{k}^{i}{)}^{⊤}h(v_{r}^{j})$$ (11)

where $u_{k}^{i}\in {\mathbb{R}}^{D\times 1}$ represents the *k*-th feature embedding of modality *i*, and $v_{r}^{j}\in {\mathbb{R}}^{1\times D}$ represents the *r*-th feature embedding of modality *j*, with *g* and *h* as learned transformations that optimize compatibility between the features of the modalities. Here, i≠j ensures cross-modal interactions, such as between CT data and clinical attributes or PET data and DNA features. To compute the attention map *P*^(*i*,*j*)^ that captures the relevance between tokens from different modalities, the following calculation is performed:


$$P^{\left(i,j\right)}={\left[\gamma_{kr}\right]}_{\left(L\times L\right)},\;\gamma_{kr}=\frac{\exp(\lambda_{kr})}{\sum_{k=1}^{L}\exp(\lambda_{kr})}$$ (12)

Each element $\gamma_{kr}$ represents the attention score between a pair of tokens from different modalities, capturing the degree of relevance between them.

Once the attention weights are calculated, these cross-modal connections are used to refine the features by aggregating relevant information across modalities. For each pair of modalities, the feature representations are updated as follows:


$$C_{i}=l(U_{i})P^{\left(i,j\right)},\;C_{j}=l(U_{j})P^{\left(i,j\right)}$$ (13)

where *C*_*i*_ and *C*_*j*_ represent the fused feature representations of each modality, and *l*(*U*_*i*_) and *l*(*U*_*j*_) denote learned transformations applied to the feature matrices *U*_*i*_ and *U*_*j*_, respectively. The resulting features are adjusted by non-negative coefficients $\beta_{i}$ and $\beta_{j}$ to preserve their individual relevance.

**Fig 6:**
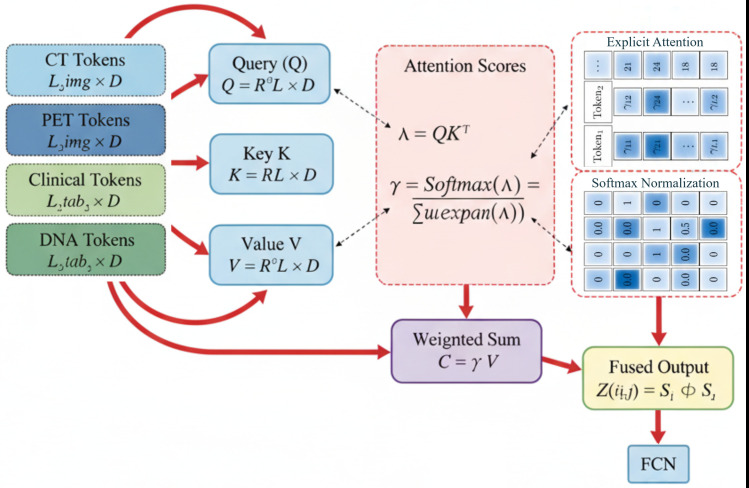
Illustration of the cross-attention mechanism for multimodal data fusion. clinical, DNA, CT, and PET data are embedded into a unified feature space. Cross-modal attention is computed between feature pairs, updating and refining modality-specific representations, which are then concatenated for further processing by the FCN in the survival prediction model.

Finally, the fused output of the cross-attention block is obtained by concatenating the refined features:


$$Z^{\left(i,j\right)}=S_{i}⊕S_{j}$$ (14)

where *S*_*i*_ and *S*_*j*_ are the enhanced, modality-specific feature maps, and ⊕ represents the concatenation operation. This cross-attention-based fusion enables the model to utilize comprehensive and interrelated feature representations from both image and tabular modalities. The output of the cross-attention, represented by the fused features *Z*^(*i*,*j*)^, is passed to the FCN phase for further processing. The FCN layer refines these multimodal features and extracts relevant patterns for survival prediction. The mechanism of cross-attention fusion is displayed in Fig 6.

### 4.6 Fully connected network phase

In this FCN phase, multimodal feature embeddings from the cross-attention module are refined into a predictive representation for survival analysis. The FCN comprises three dense layers, each progressively reducing dimensionality and capturing intricate relationships within the integrated data. Between these layers, dropout layers with a rate of 0.3 are applied to mitigate overfitting and enhance the model’s robustness on unseen data. The final dense layer produces a feature vector using linear activation, which is sent to the CoxPH model for 5-year survival prediction. This structured pipeline, illustrated under the **prediction** process in Fig 2, ensures comprehensive risk stratification by leveraging multimodal data. The approach aligns with the design principles of robust multimodal DL frameworks such as those discussed in [46,47].

### 4.7 Cox proportional-hazard workflow

The CoxPH model, a cornerstone of survival analysis, is implemented to process the feature vector derived from the FCN and to estimate 5-year survival probabilities. The hazard function is expressed as:


$$h(t)=h_{0}(t)\exp(\beta^{⊤}X),$$ (15)

where *h*_0_(*t*) is the nonparametric baseline hazard, β is the vector of learned regression coefficients, and *X* is the FCN-generated feature vector. The survival probability *S*(*t*) is computed as:


$$S(t)=\exp\left(-H_{0}(t)\exp(\beta^{⊤}X)\right),$$ (16)

With *H*_0_(*t*), the cumulative baseline hazard, estimated using methods such as the Breslow estimator, the CoxPH model is applied in a semi-parametric manner, as it does not require specific distributional assumptions for survival times, ensuring flexibility across diverse datasets [48,49]. Integrated within the CL framework, the CoxPH model dynamically adapts to new patient data while preserving critical knowledge from previous data. Techniques including EWC [37] and experience replay [50] are employed to prevent new learning from overwriting essential model parameters. For instance, when new features are incorporated, the baseline hazard *h*_0_(*t*) is recalibrated without altering existing regression coefficients β, thereby maintaining stable survival-prediction accuracy. Leveraging the PyCox library [51] (an algorithmic summary is provided in Algorithm 14), the CoxPH model is integrated with FCN-derived outputs to enable seamless operation and periodic model updates, enhancing both flexibility and adaptability in multimodal survival prediction. This implementation combines the strengths of semi-parametric modeling with dynamic CL capabilities, making it particularly effective for the multimodal survival-prediction pipeline. By continuously learning from clinical and imaging data, the framework maintains stability in previously learned parameters while sustaining consistent predictive performance over time. As illustrated in Fig 2 under the prediction section, the CL mechanism supports accurate estimation of 5-year survival probabilities while enabling robust adaptability to new incoming data.

**Algorithm 1 CL Workflow for Lung Cancer Survival Prediction**

**Require:** Dataset 𝒟 (training and new data), epochs *e*, batch size *b*, regularization strength λ

**Ensure:** Trained model and CL adaptation

1: Import necessary libraries (e.g., PyCox, Torch)

2: Load and preprocess dataset: ${𝒟}_{\text{train}}\leftarrow \text{train data},{𝒟}_{\text{new}}\leftarrow \text{new data }$

3: Initialize the CoxPH model with input dimension matching ${𝒟}_{\text{train}}$

4: Train the CoxPH model on ${𝒟}_{\text{train}}$

5: **Features:** ${𝒟}_{\text{train}}[0]$

6: **Time-to-event:** ${𝒟}_{\text{train}}[1]$

7: **Event indicator:** ${𝒟}_{\text{train}}[2]$

8: Optimize model with hyperparameters: epochs *e* = 200, batch size *b* = 32

9: Evaluate model on ${𝒟}_{\text{new}}$

10: **Compute prediction score:** $S\leftarrow \text{score}({𝒟}_{\text{new}})$

11: **Output:** print(New data score: *S*)

12: Apply CL with a hybrid method:

13: **Regularization technique:** EWC with Replay (EWC_R)

14: **Regularization strength:** λ=1000

### 4.8 Continual learning approaches

In lifelong learning, a key challenge is catastrophic forgetting, where newly acquired information overwrites previously learned knowledge. This problem becomes particularly critical in dynamic healthcare environments, where patient data are continuously updated. In lung cancer survival prediction, continual integration of clinical, imaging, and genomic data demands adaptive mechanisms that retain historical knowledge while learning from new information. Traditional replay-based methods partially address this issue by storing representative samples in a memory buffer for joint training with new data; however, as datasets grow in complexity and volume, the limited buffer capacity becomes insufficient. Thus, additional regularization mechanisms are needed to preserve important parameters and correlations learned from prior tasks.

**Fig 7:**
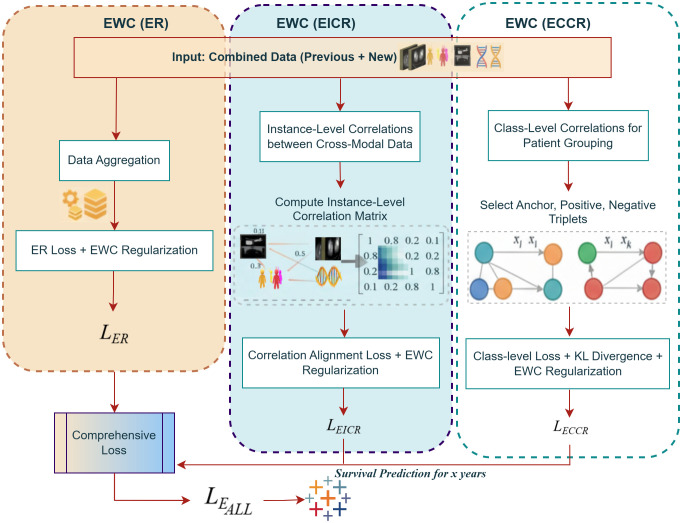
Visualization of the three EWC-based CL modules. The ER module interleaves past and new samples during training to preserve previously learned knowledge. The EICR module maintains instance-level correlations to ensure consistency for individual samples, while the ECCR module preserves class-level relationships to retain class-specific information. Together, these modules mitigate catastrophic forgetting and support stable continual learning across tasks.

**Fig 8:**
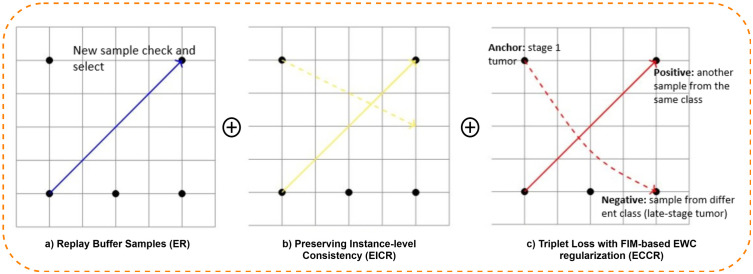
Overview of the hybrid CL mechanism. **(a)** ER maintains a fixed memory buffer (500 samples), including checking new and old samples and replays representative data; **(b)** EICR uses KL Divergence to align correlation matrices across modalities, preserving instance-level consistency; **(c)** ECCR applies Triplet Loss with FIM-based EWC regularization to stabilize class-level boundaries and avoid catastrophic forgetting.

To overcome these limitations, a hybrid CL framework is proposed that integrates replay-based mechanisms with Elastic Weight Consolidation. Specifically, the framework introduces three modules ER, EICR, and ECCR each designed to balance adaptability and retention. ER mitigates forgetting by replaying representative samples from past data alongside new instances; EICR captures inter-instance correlations across modalities to preserve fine-grained feature structures; and ECCR maintains class-level consistency through triplet-based contrastive learning. During training, newly arrived patient data are jointly processed with replayed samples, while EWC selectively constrains critical parameters to protect prior knowledge. In the prediction phase, the updated model integrates multimodal inputs, clinical, DNA, CT, and PET features via cross-attention fusion to generate 5-year survival probabilities using a FCN and CoxPH modeling. The overall CL workflow, illustrated in Fig 2, demonstrates how the memory buffer, hybrid replay strategies, and prediction modules interact. The inner workings of the three hybrid CL mechanisms are detailed in Figs 7 and 8.

### 4.9 Memory buffer management and sample selection

As illustrated in Fig 2, the proposed CL framework employs a fixed-capacity memory buffer ℳ of size *B* = 500 to mitigate catastrophic forgetting. The buffer stores representative samples from previously seen tasks and reuses them during the replay phase in conjunction with the hybrid EWC-based replay modules ER, EICR, and ECCR. The buffer operates jointly with the regularization-based mechanisms EWC [37], FIM regularization [52], and Kullback–Leibler (KL) divergence [53] to balance stability and plasticity across learning stages.

**Combined optimization objective.** During each task *t*, the total loss integrates current-task learning, replay consistency, and parameter-importance regularization as follows:


$$L=L_{new}(D_{new})+\alpha \;L_{replay}(D_{ℳ})+\beta \sum_{i}F_{i}(\theta_{i}-\theta_{i}^{old}{)}^{2}+\gamma \;KL\;(p_{old}(\cdot |x)\;\|\;p_{new}(\cdot |x)),$$

where *L*_new_ denotes the loss on current-task data *D*_new_, *L*_replay_ represents the loss on replayed samples from the buffer $D_{ℳ}$, and *F*_*i*_ corresponds to the diagonal Fisher Information weights derived from previous tasks [52]. The EWC term preserves parameter importance, while the KL divergence aligns predictive distributions between the old and updated models, ensuring output stability during replay.

**Buffer update Replay policy.** A reservoir-sampling strategy, originally proposed by Vitter [54], is adopted to maintain unbiased sample coverage across tasks. Given a stream of incoming samples, each new instance $\left(x_{i},y_{i}\right)$ is added to ℳ with probability


$$P\;((x_{i},y_{i})\in ℳ)=\frac{B}{N+i},$$

where *N* denotes the total number of samples observed so far. If the buffer is full (|ℳ|=B), one existing element is randomly replaced to preserve fixed capacity. Although reservoir sampling provides an unbiased representation of past data, it may lead to early-task dominance in small-buffer scenarios [55,56]. To address this, hybrid priority weighting from the ER, EICR, and ECCR modules is integrated to enable more balanced sample retention. By combining regularization (EWC/FIM/KL) with memory replay, this framework jointly ensures parameter-level and representation-level stability. However, effective synergy depends on balanced weighting coefficients α, β, and γ, as well as an appropriately sized buffer *B*. A small buffer may reduce replay effectiveness, insufficient regularization can cause parameter drift, and overly strong regularization may hinder new learning (plasticity). Therefore, no single mechanism is assumed to dominate; instead, the experiments confirm that their joint contribution is essential for robust continual adaptation. Detailed descriptions of each replay module are provided in the following sections.

**Interaction with hybrid replay modules.** While reservoir sampling governs sample admission, the replay modules apply distinct selection strategies when constructing replay mini-batches from ℳ:

- **ER:** Uniform random replay maintains general coverage of the past data distribution.
- **EICR:** Samples with higher instance-level correlation scores are emphasized to preserve intra-instance feature relationships.
- **ECCR:** Class-prototypical and boundary-relevant samples are prioritized to maintain inter-class consistency.

These replay mechanisms operate jointly with the EWC–FIM–KL regularization terms to preserve both feature- and parameter-level knowledge throughout training. Detailed formulations for ER, EICR, and ECCR are provided in the following sections.

### 4.10 EWC experience replay module

The ER module forms the foundation of the CL framework by combining memory-based rehearsal with EWC. This hybrid approach ensures that the model continuously learns new information while retaining previously acquired knowledge without catastrophic forgetting, as visualized in Fig 7.

A replay memory buffer is established to store representative samples from earlier training phases. In this design, the buffer size is fixed at 500 samples. This value is chosen as a trade-off between:

- maintaining sufficient variety to represent different classes and modalities, and
- limiting GPU memory overhead for multimodal data (CT, PET, clinical, and DNA).

Each time new data is introduced, a subset of these 500 stored samples is mixed with current samples to form the next training batch. This ensures that the model always “rehearses” old knowledge alongside learning new patient data, thereby maintaining temporal consistency [16].

During training, ER retrieves replay samples and combines them with new task data in a unified loss function that merges data-based rehearsal with parameter-based regularization:


$$L_{ER}={𝔼}_{(x,y)∼M_{t}}[\ell (y,f_{\theta }(x))]+\lambda \sum_{i}F_{i}(\theta_{i}-\theta_{i}^{*}{)}^{2}$$ (17)

Each component in this loss plays a distinct role: – ${𝔼}_{(x,y)∼M_{t}}[\ell (y,f_{\theta }(x))]$: The expected task loss (cross-entropy or survival regression loss) computed on mixed batches containing both new and replayed samples (*x*, *y*). It enforces instance-level retention by maintaining predictive consistency on previously seen data. - λ: A regularization coefficient that balances the trade-off between adaptation (plasticity) and retention (stability). - *F*_*i*_: The FIM value for parameter $\theta_{i}$, representing how critical that parameter is for prior tasks. - $(\theta_{i}-\theta_{i}^{*}{)}^{2}$: The squared deviation of the current parameter $\theta_{i}$ from its previous optimal value $\theta_{i}^{*}$, penalizing large updates to important weights. At the end of each task, the Fisher Information [57] for each parameter is computed as:


$$F_{i}={𝔼}_{x∼D_{t}}\left[{\left(\frac{\partial \log p(y|x,\theta )}{\partial \theta_{i}}\right)}^{2}\right]$$

Here: - *D*_*t*_: Dataset of the current task *t*, – p(y|x,θ): Model’s likelihood of predicting the correct outcome, – $\frac{\partial \log p(y|x,\theta )}{\partial \theta_{i}}$: Gradient indicating how sensitive the likelihood is to parameter $\theta_{i}$. Parameters with larger *F*_*i*_ values are deemed more influential and are penalized more strongly during updates in future tasks. The batch construction dynamically mixed newly acquired and replayed data, ensuring consistent representation across all training sessions. This balanced loss and replay strategy significantly reduced performance degradation during task transitions, demonstrating the efficacy of this module in retaining both local and global feature patterns. The ER module successfully enables continuous learning while maintaining robust survival-prediction capability for lung cancer patients. This hybrid approach ensured the integration of historical and new knowledge, addressing the complexities of multimodal datasets. As supported by recent works [58–60], combining replay-based rehearsal with Fisher-weighted regularization provides a stable–plastic balance ideal for lifelong medical learning scenarios.

### 4.11 EWC instance-level correlation replay module

The EICR module is designed to preserve fine-grained cross-modal dependencies between multimodal inputs such as CT, PET, clinical, and DNA features. While the ER module primarily retains individual instances through memory replay, the EICR module extends this concept by aligning relational structures among samples to ensure that learned inter-instance correlations are not lost when new data are introduced. As illustrated in Fig 7, the process includes correlation matrix computation, alignment of correlation structures between previous and current learning phases, and parameter regularization through EWC.

To achieve this, a correlation matrix $C\in {\mathbb{R}}^{n\times n}$ was constructed to model pairwise relationships between feature embeddings within each training batch. Let $F=\{f_{1},f_{2},\ldots ,f_{n}\}$ denote the feature set extracted from multimodal encoders (SwinT for imaging, XLNet for DNA, and FCN for clinical data). The correlation transformation is defined as:


$$\phi :F\to C\in {\mathbb{R}}^{n\times n},$$ (18)

$$C_{ij}=\varphi (f_{i},f_{j}),\;C_{ij}\in \mathbb{R},$$ (19)

where φ computes the pairwise similarity between two feature embeddings *f*_*i*_ and *f*_*j*_. To capture non-linear dependencies across modalities, φ is approximated using a high-order Taylor expansion of the Gaussian Radial Basis Function (RBF) [61]:


$$\varphi (f_{i},f_{j})=\exp(-\gamma \|f_{i}-f_{j}{\|}^{2})\approx \sum_{p=0}^{P}\frac{\exp(-2\eta )}{2\eta^{p}\;p!}(f_{i}\cdot f_{j}^{T}{)}^{p},$$ (20)

where η controls correlation smoothness, and *p* defines the expansion order. This formulation enables high-order representation of complex relationships, for instance, DNA mutation clusters correlated with PET-based tumor activity or clinical progression markers. To prevent the loss of learned relational patterns across incremental updates, the correlation structures from the previous task (*C*^*P*^) and the current task (*C*^*C*^) are aligned using a hybrid EICR loss:


$$L_{EICR}={𝔼}_{f_{\theta }(x)∼M_{t}}\left(\frac{1}{2}\|C^{C}-C^{P}{\|}^{2}\right)+\lambda \sum_{i}F_{i}(\theta_{i}-\theta_{i}^{*}{)}^{2}.$$ (21)

The first term represents the Frobenius norm between correlation matrices, preserving pairwise alignment and preventing structural drift at the instance level [62]. This ensures that relationships such as “mutation–tumor intensity” or “clinical score–lesion size” remain stable across learning phases. The second term is the EWC regularization, where *F*_*i*_ represents the FIM [57], capturing the importance of each parameter, and $\theta_{i}^{*}$ denotes its previously optimal value. The coefficient λ balances the trade-off between correlation alignment and plasticity during adaptation.

During implementation, both correlation matrices and key sample embeddings were stored in the replay buffer. Each time new data were introduced, their pairwise correlation structures were compared against previously stored patterns to maintain consistency. In this process, the Kullback–Leibler (KL) divergence was used as an auxiliary measure to assess divergence between prior and current correlation distributions, ensuring that structural drift remained minimal [58,59]. This technique allowed the model to dynamically correct deviations between old and new representations, promoting smoother transitions during incremental learning. As a result, the EICR module ensures that the model does not simply memorize instances but also retains the relational geometry among multimodal representations. This instance-level correlation preservation is essential for maintaining clinically meaningful dependencies such as mutation-driven phenotype changes across evolving datasets. The incorporation of KL divergence alignment and EWC-based parameter regularization together provides an adaptive mechanism that retains structure and stability while accommodating new patient data. This capability is particularly crucial in medical CL, where relational consistency often underpins accurate disease progression modeling [60,63]. By leveraging the EICR module, the framework adapts effectively to new datasets while retaining essential information for accurate survival predictions in lung cancer patients.

### 4.12 EWC class-level correlation replay module

In this stage of the experiments, the ECCR module is developed to preserve class-specific knowledge across different patient groups, ensuring consistent survival prediction for categories such as early-stage and late-stage cancer patients. To achieve this, EWC is combined with a triplet-based correlation mechanism tailored to the multimodal dataset, which includes CT, PET, clinical, and DNA data. This approach enables the maintenance of class-level distinctions while incorporating new data without catastrophic forgetting [37]. A triplet mechanism is implemented in which, for each anchor *x*_*i*_ (e.g., a patient with early-stage cancer), a positive sample *x*_*j*_ (another early-stage patient) and a negative sample *x*_*k*_ (a late-stage patient) are selected, as illustrated in Fig 7. This setup preserves both intra-class similarities and inter-class differences. For example, CT and PET tumor-intensity patterns serve as anchor–positive pairs within the same class, while DNA mutations or clinical time-series features help differentiate anchor–negative pairs. The triplet loss is computed as follows:


$${\text{Dist}}_{\phi }(x_{i},x_{j})=\|\phi (x_{i})-\phi (x_{j}){\|}^{2},\;{\text{Dist}}_{\phi }(x_{i},x_{k})=\|\phi (x_{i})-\phi (x_{k}){\|}^{2},$$ (22)

where ϕ(x) represents the fused feature embedding obtained through cross-attention layers processing data from all modalities. To ensure meaningful class-level separation, a semi-hard triplet selection strategy is applied, in which negative samples *x*_*k*_ are chosen such that:


$${\text{Dist}}_{\phi }(x_{i},x_{j})<{\text{Dist}}_{\phi }(x_{i},x_{k})<{\text{Dist}}_{\phi }(x_{i},x_{j})+\epsilon ,$$ (23)

with ϵ defining the margin. For example, genomic variations were used to find samples that were similar but not identical within the same class. In this implementation, the triplet mechanism is complemented with a probabilistic alignment strategy. For each triplet, the class-level probability distribution is computed as:


$$p_{ijk}(\phi )=\frac{\exp(-{\text{Dist}}_{\phi }(x_{i},x_{j})/\tau )}{\exp(-{\text{Dist}}_{\phi }(x_{i},x_{j})/\tau )+\exp(-{\text{Dist}}_{\phi }(x_{i},x_{k})/\tau )},$$ (24)

where τ is the temperature parameter. This probabilistic distribution helped us measure how well class separability was maintained across training updates. To align prior and current training phases, the Kullback–Leibler divergence is minimized between the Bernoulli distributions derived from past (*P*^*P*^) and current (*P*^*C*^) class-level probabilities [53]:


$$L_{ECCR}={𝔼}_{(x_{i},x_{j},x_{k})∼M_{t}}\left[\sum_{ijk}{\text{D}}_{KL}(P_{ijk}(\phi^{P})∥P_{ijk}(\phi^{C}))\right].$$ (25)

This loss ensures the preservation of key class-level patterns during training. In the proposed framework, the ECCR loss is combined with EWC to protect critical weights from past tasks:


$$L_{EWC}=\lambda \sum_{i}F_{i}(\theta_{i}-\theta_{i}^{*}{)}^{2},$$ (26)

where $\theta_{i}$ and $\theta_{i}^{*}$ represent the current and prior weights, respectively, and *F*_*i*_ denotes fisher information for each parameter.

Finally, the combined objective function for training included the survival prediction loss *L*_CoxPH_, along with replay and regularization terms:


$$L_{\text{All}}=L_{\text{CoxPH}}+\alpha L_{EWC}+\beta L_{ER}+\gamma L_{EICR}+\delta L_{ECCR},$$ (27)

where α, β, γ, and δ are hyperparameters that balance the contributions of each term. Specifically: – *L*_*ER*_ represents the ER loss for direct replay of past data. - *L*_*EICR*_ is the instance-level correlation replay loss. - *L*_*ECCR*_ is the class-level correlation replay loss, as described above in the hybrid loss function. The experimental setup involves extracting multimodal features from CT and PET images using a SwinT, which captures spatial and intensity-based tumor characteristics. DNA data, including mutation metrics, was processed using XLNet to learn latent genomic features, while clinical data, such as lab records and vital signs, was modeled using FCN networks to capture temporal patterns. These features were fused using a cross-attention mechanism to create a unified embedding. The embeddings were then processed through the ECCR, ER, and EICR modules, where the ECCR ensured class-level correlation learning by maintaining intra-class similarity and inter-class dissimilarity. The output of these modules was passed to the FCN, which further processed the learned features. Finally, during the prediction phase, the model used the CoxPH loss function (*L*_CoxPH_) to compute survival probabilities, with guidance from the hybrid loss function. The integrated mechanisms of ER, EICR, and ECCR played critical roles in preserving important patterns from previous tasks while enabling the incorporation of new patients information [54]. By preserving class-level relationships and aligning past and current knowledge through EWC-based regularization [37], the model ensures that it does not forget previously learned survival patterns while adapting to new data. This process enables the model to predict the 5-year survival probability for each patient, with stable and accurate predictions over time.

### 4.13 Comprehensive explanation of CL framework

The CL component of the framework operates as an adaptive mechanism that enables the model to incorporate new patient data while retaining previously learned information. The complete process is illustrated in Fig 2, which shows the flow from base model training to incremental learning and the three replay-enhanced mechanisms (ER, EICR, ECCR). This section provides a detailed explanation of how input data, memory replay, and knowledge regularization interact during training as shown in Fig 8.

**Input and Base Model Training:** The training begins with multimodal data inputs, including CT, PET, clinical, and DNA features. Each modality is encoded separately: SwinT extracts spatial features from CT and PET images, XLNet learns contextual genomic patterns from DNA, and an FCN embeds the clinical data. These representations are fused through a cross-attention block to produce a shared multimodal embedding, which is then sent through FCN and trained using the CoxPH loss for survival prediction:


$$L_{CoxPH}=-\sum_{i}\delta_{i}\left(\beta^{T}x_{i}-\log\sum_{j\in R_{i}}e^{\beta^{T}x_{j}}\right)$$

where $\delta_{i}$ denotes the event indicator and *R*_*i*_ is the risk set. This establishes a strong base model before CL begins.

**Continual Learning Stage:** Once the base model is trained, new patient data streams are introduced incrementally. The CL pipeline processes this new information through three mechanisms: ER, EICR, and ECCR, each addressing different aspects of catastrophic forgetting.

**Experience Replay:** ER acts as the foundation for maintaining historical knowledge. A fixed memory buffer (set to 500 representative samples) stores key patient instances selected through balanced sampling, ensuring coverage of diverse classes and modalities. When new data arrive, ER performs a similarity check between current and stored samples using feature embeddings. This helps decide which samples to replay and which to replace in the buffer. During training, ER merges new data with stored samples and computes a joint loss with EWC regularization:


$$L_{ER}={𝔼}_{(x,y)∼M_{t}}[\ell (y,f_{\theta }(x))]+\lambda \sum_{i}F_{i}(\theta_{i}-\theta_{i}^{*}{)}^{2}$$

where *M*_*t*_ is the replay memory, and *F*_*i*_ is the FIM capturing parameter importance. This penalizes deviation from previous task-critical weights while allowing new parameters to adapt, balancing plasticity and stability (see Fig 8(a)).

**Instance-Level Correlation Replay:** EICR captures fine-grained relationships between patient instances across modalities. When new data are introduced, the correlation matrix of multimodal embeddings (e.g., between CT–PET or DNA–clinical pairs) is recomputed and compared against the previous correlation state as exhibit in Fig 8(b). This alignment is performed through Kullback–Leibler (KL) Divergence [64]:


$$L_{EICR}=D_{KL}(P∥Q)+\lambda \sum_{i}F_{i}(\theta_{i}-\theta_{i}^{*}{)}^{2}$$

where *P* and *Q* represent instance-level correlation distributions before and after the update. The KL term minimizes distribution drift, ensuring that newly added patients do not distort previously learned inter-instance dependencies. This mechanism prevents “pattern drift,” a common issue in medical data streams, as discussed in recent CL studies [58,59].

**Class-Level Correlation Replay:** ECCR enhances global class boundary retention by leveraging triplet sampling consisting of an anchor, a positive sample from the same class (e.g., early-stage tumor), and a negative sample from a different class (e.g., late-stage tumor). This triplet-based training encourages intra-class compactness and inter-class separability:


$$L_{ECCR}=L_{triplet}+\lambda \sum_{i}F_{i}(\theta_{i}-\theta_{i}^{*}{)}^{2}$$

Here, the FIM term stabilizes parameter updates that are vital for class separation, reducing boundary collapse. As shown in Fig 8(c), dashed paths represent how positive and negative relationships are reinforced or penalized through the triplet loss. By combining triplet optimization with EWC, ECCR ensures that newly introduced class information does not overwrite learned distinctions, maintaining robustness in class-level knowledge consolidation.

**Overall Integration:** The total CL objective integrates all three replay losses with weighted regularization:


$$L_{EAll}=L_{CoxPH}+\alpha L_{ER}+\beta L_{EICR}+\gamma L_{ECCR}$$

This unified loss ensures that ER handles long-term memory, EICR preserves cross-modal consistency, and ECCR stabilizes class representations. The combination enables balanced adaptation and retention, effectively reducing forgetting while improving survival prediction accuracy.

Recent CL research such as [58,59,65] emphasizes that hybrid approaches combining parameter-based (EWC) and memory-based (replay) mechanisms achieve the most reliable stability–plasticity balance. The proposed ER–EICR–ECCR design follows this principle by merging Fisher-based importance weighting, distributional alignment, and relational replay to create a dynamic, adaptive survival-prediction system.

### 4.14 Ethics statement

This study utilised the publicly available AI-Hub Lung Cancer Multimodal Dataset [66], which contains de-identified clinical, imaging (CT, PET), and genomic data collected under national ethical approval and informed consent procedures in South Korea.

All data were anonymised prior to public release, and no identifiable personal information was accessed by the authors. As the study involves secondary analysis of fully anonymised public data, additional institutional review board (IRB) approval was not required.

## 5 Experimental Setup

**Dataset:** A comprehensive multi-institutional dataset provided by AI Hub [66], comprising records of **5,053 lung cancer patients**, is utilized in this study. Among these, **3,770 patients** had complete multimodal information, including clinical, CT, PET, and DNA mutation data, along with survival outcomes spanning up to four years. However, DNA mutation data were available for only **412 patients**. To address this sparsity, we employed **XLNet**, a transformer-based sequence model, to learn contextual patterns from the available DNA data. Instead of direct supervised training, XLNet is pretrained to generate latent representations of mutation features, which are then integrated as embeddings into the multimodal survival prediction framework alongside clinical, CT, and PET modalities.

For evaluation, the dataset was organized into three subsets to simulate continual data acquisition. The primary training set (**D_3358P**) included 3,358 patients with complete clinical, CT, PET, and XLNet-derived DNA embeddings. Two additional cohorts **D_200P** (200 patients) and **D_212P** (212 patients) containing real DNA mutation data were used to emulate incremental updates in CL scenarios. This design enabled systematic assessment of the framework’s adaptability to new patient data while preserving prior knowledge.

**Clinical Data Preprocessing:** Clinical data contained **16 features** describing patient demographics, disease characteristics, and lifestyle attributes, including PatientID, Gender, Age, Survival.time, Deadstatus.event, Overall.stage, and TNM staging (T, N, M). Lifestyle factors included Smoking.status and Smoking.amount. Data preprocessing involved several standardized steps: (i) Outlier detection and removal using the Z-score method (|*z*| > 3), (ii) One-hot encoding for categorical variables (e.g., gender, smoking status), (iii) Min–max normalization to ensure consistent feature scaling, and (iv) Elimination of redundant or non-informative columns (Mcode.description, Histology, and FILE_NAME). The primary targets for survival modeling were Survival.time and Deadstatus.event. Missing values were handled via mean imputation for continuous variables and mode imputation for categorical attributes to maintain dataset integrity. The cleaned and normalized clinical data were subsequently passed to a FCN for feature embedding.

**CT/PET Data Preprocessing:** To ensure uniformity across imaging modalities, a standardized 3D preprocessing pipeline for CT and PET scans is designed before input into the SwinT. DICOM volumes were sorted according to their SliceLocation metadata to preserve anatomical order, followed by resampling to a fixed spatial resolution of 128×128 pixels and a depth of 160 slices. For scans with fewer slices, zero-padding was applied (background intensity: −2000 HU for CT and 0 for PET); for scans exceeding the target depth, cubic spline interpolation was used to maintain anatomical consistency. The resulting volumes were formatted as [1, 160, 128, 128] tensors suitable for SwinT input. Data augmentation included random horizontal/vertical flips, ± 10° rotations, and Gaussian noise addition to enhance generalization. Intensity normalization was performed to a [0,1] range. The dataset was divided into training, validation, and test sets (80%, 10%, 10%), ensuring representative class balance. Preprocessed 3D volumes were stored in NumPy format for efficient reuse during training.

**Fig 9:**
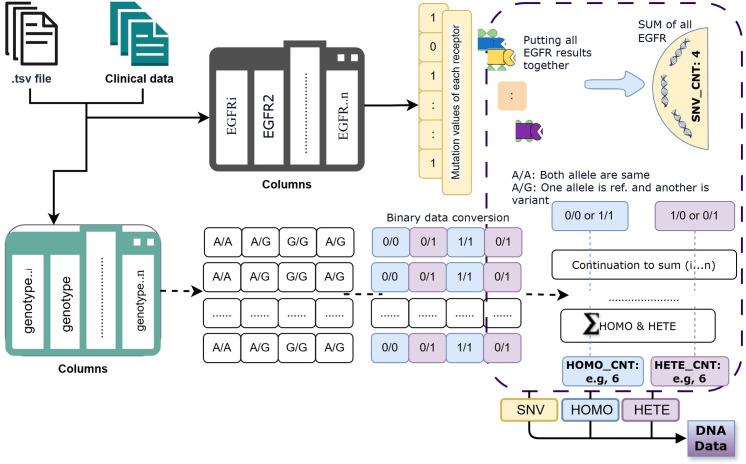
An overview of the DNA preprocessing workflow, where patient-specific .tsv files containing genotype data (e.g., A/A, A/G, G/G) are converted into binary representations (0/0, 0/1, 1/1) to identify HOMO and HETE variants. Aggregated counts of SNV, HOMO, and HETE are computed across receptors and normalized to form structured DNA features for XLNet-based embedding and multimodal integration.

**DNA Data Preprocessing:** DNA mutation data were provided as patient-specific .tsv files containing variant-level genomic annotations. Each file recorded mutation TYPE and ZYGOSITY information (i.e., HOMO or HETE). The preprocessing workflow consisted of: (1) Reading and concatenating all patient-level files into a unified DataFrame; (2) Cleaning invalid or missing entries in TYPE or ZYGOSITY columns; (3) Replacing empty files with patient-wise mean values to preserve data continuity; and (4) Extracting three aggregate mutation metrics per patient counts of **SNVs**, **HOMO variants**, and **HETE variants**. These metrics capture essential tumor-level genomic variation and heterogeneity. After feature extraction, values were min–max normalized to [0,1] for cross-modality comparability. The resulting structured DNA data were saved in a summary matrix and used to pretrain XLNet for latent representation learning of mutation patterns. Figs 9 and 4 illustrates the DNA preprocessing and training pipeline, including data extraction, cleaning, normalization, and XLNet embedding generation for integration into the multimodal CL framework.

**Data Alignment Across Modalities:** All preprocessing pipelines were synchronized using PatientID as a unique identifier to ensure accurate multimodal alignment. Each modality clinical, imaging, and genomic was linked to its corresponding patient record, preventing data leakage or mismatch. During continual learning updates, new patient data followed the same preprocessing pipeline, ensuring seamless integration into the existing framework.

**Pre-training Setting:** All experiments were conducted using a default pre-training configuration of 200 epochs unless specified otherwise. To optimize model performance, the AdamW optimizer was employed with a batch size of 32, ensuring efficient weight updates and regularization. The implementation was carried out using the PyTorch framework, leveraging its flexibility for model design and training. The computational setup included 2 NVIDIA GeForce RTX 3070 GPUs, 64 GB of RAM, and an Intel(R) i9-10900 processor, providing sufficient resources for handling complex computations and ensuring smooth training. This configuration was chosen to balance efficiency and scalability while maintaining consistent results across investigations.

**Evaluation Setting on Survival Prediction:** To assess the performance of the CL-based multimodal framework, two widely recognized survival-prediction metrics are employed: the Concordance Index and the Mean Absolute Error. The C-index measures predictive accuracy by quantifying the proportion of correctly ordered survival pairs, i.e., whether patients with shorter survival times are predicted with higher risk scores [67]. It is computed as:


$$C\text{-index}=\frac{1}{\left|𝒫\right|}\sum_{(i,j)\in 𝒫}1[({\hat{y}}_{i}>{\hat{y}}_{j})\wedge (t_{i}<t_{j})],$$ (28)

where 𝒫 is the set of comparable patient pairs, $\hat{y}$ is the predicted risk score, and *t* denotes observed survival time. The MAE quantifies the absolute deviation between predic*t*ed and true survival times:


$$MAE=\frac{1}{N}\sum_{i=1}^{N}|t_{i}-{\hat{t}}_{i}|,$$ (29)

where *N* is the number of patients, *t*_*i*_ the true time, and ${\hat{t}}_{i}$ the predicted survival time [68].

For CL evaluation, four standard metrics were adopted [37,58]:


$$\text{Baseline Performance (BP)}=A_{0},\;\text{New Data Performance (NDP)}=A_{t},$$ (30)

where *A*_0_ and *A*_*t*_ represent accuracy or C-index on the initial and latest datasets, respectively. Knowledge Retention (KR) and Forgetting (Fg) over tasks and updates are also reported.

Let $A_{t}^{\text{before}}$ denote performance on prior data before learning update *t*, and $A_{t}^{\text{after}}$ performance on the same prior data after learning upda*t*e *t*. With *T* total updates,


$$KR\;=\;\frac{1}{T}\sum_{t=1}^{T}\frac{A_{t}^{\text{after}}}{A_{t}^{\text{before}}}.$$ (31)

Lower values indicate better resistance to forgetting. Following standard CL practice, the metric is defined as:


$$Fg\;=\;\frac{1}{T-1}\sum_{t=2}^{T}\max_{k\in [1,\;t-1]}(A_{k}^{\text{before}}-A_{t}^{\text{after}}{)}_{+},\;(x{)}_{+}=\max(x,0).$$ (32)

Lower *Fg* and higher *KR* indicate better long-term knowledge preservation and reduced catastrophic forgetting. To ensure statistical rigor, each experiment was repeated three times using different random seeds, and the mean and standard deviation of all metrics were reported. Ninety-five percent confidence intervals (95% CI) were calculated based on the patients *t*-distribution to account for sampling variability. Statistical significance between competing methods was evaluated using paired *t*-tests, where results with *p* < 0.05 were considered statistically significant. In addition, ablation studies were performed to quantify the contribution of each modality and the cross-attention fusion mechanism within the CL workflow, providing a deeper understanding of each component’s impact on overall survival prediction performance.

## 6 Results and empirical evaluation

### 6.1 Ablation studies

In the ablation study, the performance of the proposed CL framework for lung cancer survival prediction is evaluated by incrementally modifying its core components. These experiments examined the contribution of key strategies, including EWC, replay-based mechanisms, cross-attention fusion, and the inclusion of DNA embeddings. To ensure fair comparisons, all experiments were conducted with identical hyperparameters: a batch size of 32, the AdamW optimizer, a learning rate of $1\times 10^{-4}$, and a weight decay of $1\times 10^{-4}$. During the ablation analyses, the **baseline training dataset (***D*_3358*P*_**)** is used together with two CL evaluation cohorts *D*_200*P*_ and *D*_212*P*_ to simulate incremental data updates. Metrics were explicitly computed for each dataset split: *training*, *validation*, and *CL evaluation*. The evaluation metrics include the C-index, MAE in days, KR, and Fg, as summarized in Table 1. To ensure transparency and reproducibility, we provided a detailed mapping between each reported result and its corresponding configuration file, experimental script, and dataset split used in generating the results.

**Table 1:** Ablation Study for Lung Cancer Survival Prediction Framework.

Datasets Used:	D_3358P, D_200P, and D_212P			
Model Variant	C-Index (↑)	MAE (Day ↓)	KR	Fg (↓)
**HCLmNet (Proposed)**	**0.84**	**140**	**0.86**	**0.08**
**× CL Mechanism**	0.78	180	0.25	0.83
**× DNA Modality**	0.81	155	0.66	0.26
**Raw DNA Features → XLNet**	0.79	260	0.64	0.19
**Concatenation → Cross-Attention**	0.80	168	0.65	0.35
**CL Replay → EWC**	0.82	155	0.63	0.16
**CL EWC → Replay**	0.83	190	0.71	0.45

**Notes:** – C-Index (↑): Higher values indicate better discriminative ability. - MAE (↓): Lower values reflect higher accuracy in survival time prediction. - Knowledge Retention (KR): Performance retention metric post-training with new data. - Forgetting (Fg): Measures performance degradation on old data.

**HCLmNet (Proposed):** The full framework integrates a FCN for clinical, SwinT for CT/PET feature extraction, XLNet for generating DNA embeddings, cross-attention for multimodal fusion, and CoxPH for survival prediction. Combined with both EWC and replay mechanisms, this configuration achieves the best results, with a C-Index of 0.84, MAE of 140 days, KR of 0.86, and minimal Fg at 0.08. The superior performance underscores the importance of integrating all these elements for effective survival prediction.

**Without CL Mechanism:** Eliminating both EWC and replay mechanisms leaves the model susceptible to catastrophic forgetting, resulting in degraded performance. The C-Index drops to 0.78, MAE increases to 180 days, and KR decreases to 0.25, while Fg increases to 0.83. This highlights the critical role of CL in preserving knowledge during incremental updates.

**Without DNA Modality:** To assess the impact of input data diversity, the DNA modality is removed. Then the framework processes clinical, CT and PET features only. Consequently, the C-Index falls to 0.81, MAE rises to 155 days, and KR drops significantly to 0.66, with Fg increasing to 0.26. These results emphasize the significance of DNA data in improving predictive accuracy.

**Embedding with Raw DNA Features:** Here, the XLNet embeddings replaced with raw DNA mutation features eliminate pretrained DNA representations, leading to poorer performance. The C-Index decreases to 0.79, MAE increases sharply to 260 days, KR drops to 0.64, and Fg rises to 0.19. This demonstrates the advantages of leveraging advanced integrating for DNA data.

**Using Concatenation Instead of Cross-Attention:** In this variant, cross-attention is replaced with feature concatenation, which removes the dynamic alignment and weighting of multimodal inputs. The resulting C-Index is 0.80, MAE increases to 168 days, and KR drops to 0.65, with Fg increasing significantly to 0.35. The findings underline the superiority of cross-attention for multimodal fusion.

**CL Replay method apply Instead of EWC:** In this configuration, the framework retains only the replay mechanism, relying on a memory buffer to revisit past examples during training. However, without EWC, which regularizes parameters by preserving critical weights, the model becomes susceptible to information leakage, where significant features from earlier tasks are not adequately preserved in subsequent updates. This results in incomplete learning dynamics, as the loss function struggles to balance between past and new data. Consequently, the model achieves a C-Index of 0.82, MAE of 155 days, KR of 0.63, and Fg of 0.16. While the replay mechanism demonstrates the ability to mitigate forgetting to some extent by reintroducing prior data, it lacks the parameter-level protection that EWC provides, leading to suboptimal retention of old information. This highlights the importance of combining replay with other CL techniques for robust knowledge preservation and minimal catastrophic forgetting.

**CL EWC method apply Instead of Replay:** In this setting, EWC is applied as the sole CL mechanism, removing the replay buffer entirely. EWC works by introducing a parameter regularization term in the loss function, which penalizes deviations from previously important weights. This helps retain critical features of earlier tasks by anchoring the model parameters to previously learned distributions. Due to that, the model achieves a C-Index of 0.83, MAE of 190 days, KR of 0.71, and Fg of 0.45. While EWC effectively minimizes forgetting by preserving parameter stability, its performance is limited in scenarios with significant distribution shifts in incoming data. Without replay, the model lacks the ability to refresh its understanding of earlier data, which can lead to over-reliance on parameter constraints and a higher MAE (see Table 1). This highlights the complementary nature of replay and EWC: while EWC stabilizes parameters for knowledge retention, replay reinforces memory by revisiting earlier examples, ensuring a more comprehensive learning process. The results emphasize the importance of combining these techniques to balance robust parameter protection and dynamic memory reinforcement, particularly in complex multimodal learning tasks.

**Fig 10:**
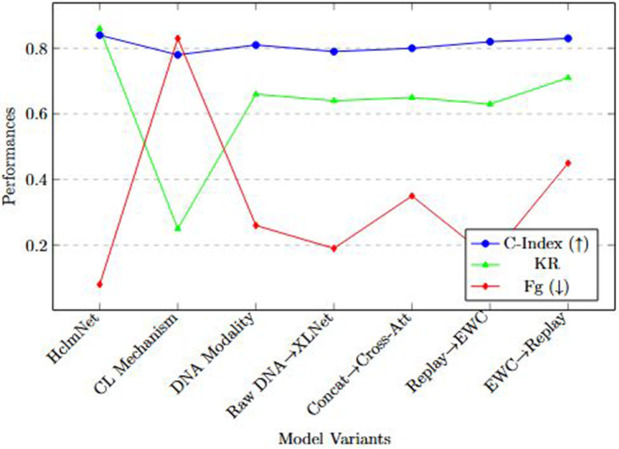
Ablation Study Results: Comparison of C-Index, KR, and Fg Across Variants. The proposed HCLmNet improves the C-Index by 7.7%, demonstrating better learnability. KR rises from 0.25 to 0.86, and Fg drops significantly by 90%, highlighting the effectiveness of combined replay and EWC strategies. Removing DNA modality and cross-attention notably affects performance, underscoring their importance.

This ablation study highlights the critical role of each component within the CL framework, underlining the significance of combining various mechanisms to achieve optimal performance.The full framework integrating EWC, replay, cross-attention fusion, XLNet-learned DNA embeddings, and CoxPH consistently outperforms all other model variants across key metrics, as visualized in Fig 10. Specifically, the inclusion of EWC and replay ensured a strong balance between knowledge retention and minimizing forgetting, while the cross-attention mechanism facilitated effective interaction between modalities. The integration of DNA embeddings contributed to the model’s ability to capture complex patterns from genetic data, strengthening its overall performance. In contrast, removing any of these components led to a noticeable degradation in model performance. For example, excluding the replay mechanism or using raw DNA features instead of embeddings resulted in higher forgetting and decreased knowledge retention, as observed in the increased MAE and reduced C-Index scores. These findings reinforce the importance of each individual design choice and demonstrate how the synergy of all techniques enhances the framework’s capability for incremental learning. Ultimately, the ablation study validates the approach, demonstrating its robustness in retaining previously learned knowledge while adapting to new data without significant degradation in performance.

### 6.2 Experimental results and analysis

**Fig 11:**
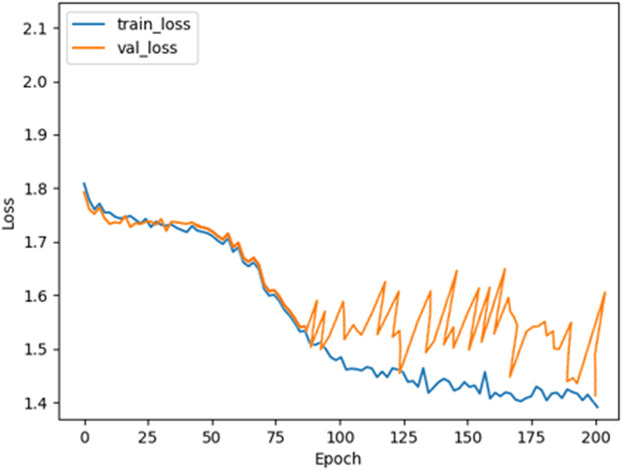
Baseline Model: Training and Validation loss over epochs.

A comprehensive evaluation of the lung cancer survival prediction framework is conducted using three datasets: **D_3358P** for training and evaluating the base model, and **D_200P** and **D_212P** for evaluating the CL pipeline. The base model was initially trained on the **D_3358P** dataset for 200 epochs using a batch size of 32, an Adam optimizer, a learning rate of $1\times 10^{-4}$, and a weight decay of $1\times 10^{-4}$. It achieved a training loss of **0.894** and a validation loss of **1.259**, as visualized in Fig 11. This indicates the model’s ability to identify patterns effectively during training, demonstrating convergence and stability.

**Fig 12:**
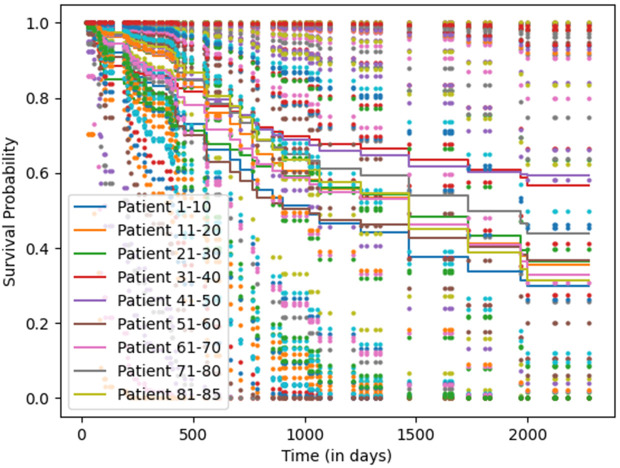
Baseline Evaluation: Survival probability trends over 5 years for 86 Patients. The plot shows the probability for 86 lung cancer patients over 5 years. Each point represents an individual patient’s survival prediction at specific time intervals, with distinct colors for each patient. The plot illustrates a realistic decline in survival probability over days.

Subsequently, the base model was evaluated on the **D_200P** dataset, comprising diverse lung cancer patients with varying ages and cancer subtypes. The evaluation yielded a **C-Index of 0.7656** and an **MAE of 189.4293**, which are reasonable given the complexity of predicting 5-year survivability. The survival probability graph in Fig 12, reflects this capability, showing a natural decline in survivability as the timeline progresses. This trend aligns with clinical observations, highlighting the model’s predictive reliability for a general lung cancer cohort. The evaluation thus validated the base model’s capability to generalize well across unseen data.

**Fig 13:**
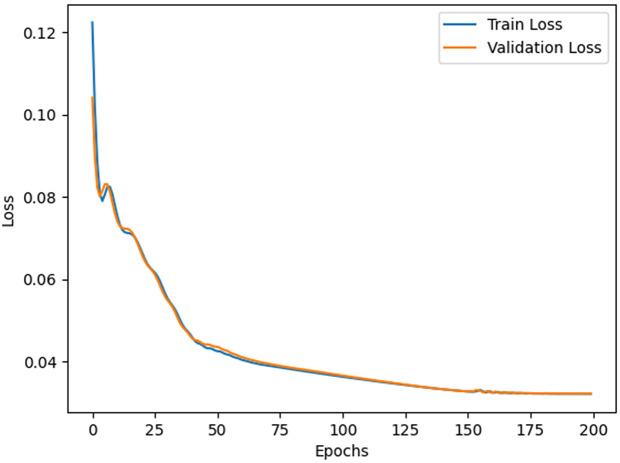
CL Model: Training and Validation loss over epochs.

To analyze the effectiveness of the CL framework, two incremental datasets: **D_200P** and **D_212P** were introduced. These datasets were incorporated sequentially into the pipeline to simulate real-world scenarios where new patient data continuously becomes available. The incremental learning module was designed to preprocess incoming data and leverage CL strategies to assimilate this knowledge while preserving previously learned patterns. The model underwent an additional 200 epochs of training on the combined dataset using the same configuration as the base training phase. This resulted in significantly improved metrics, with the training loss reduced to **0.0339** and the validation loss to **0.0324** (Fig 13). These low loss values suggest the model effectively adapted to new information without overfitting or degradation in performance.

**Fig 14:**
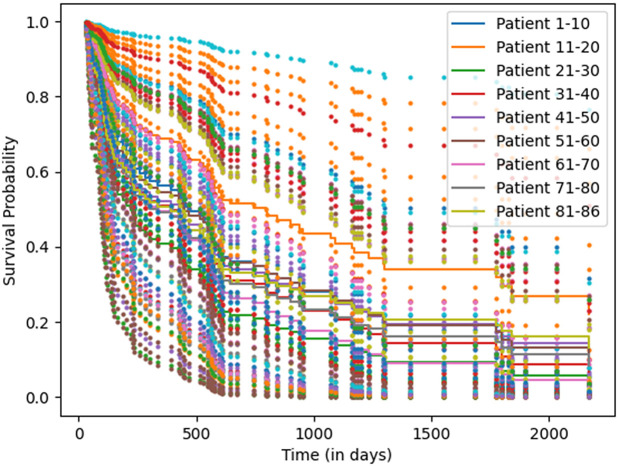
CL Evaluation: Survival probability trends Over time for 86 patients. The plot presents survival probability direction for 86 lung cancer patients, tracked over a period of 5 years. Each data point represents the survival prediction for an individual patient at specific intervals, with distinct colors indicating each patient’s unique trend. As new data is introduced and the model is trained with both previous and new data, the resulting evaluation shows smoother survival trajectories with reduced variability. This illustrates the effectiveness of the CL strategies in accurately predicting survival outcomes while minimizing noise and ensuring consistency across patient-specific survival paths.

In terms of predictive metrics, the HCLmNet framework achieves a **C-Index of 0.8456** and an **MAE of 140.4233**, demonstrating improved accuracy and discriminative ability compared to the base model. The survival-prediction graphs plotted for patients over a 5-year timeline exhibit smoother and more realistic trajectories, with reduced variance between predicted probabilities and expected outcomes. This improvement is evident from Fig 14, where the survival probabilities for different patient groups more accurately align with clinical expectations. The CL framework effectively mitigated forgetting, preserved knowledge retention, and adapted to new data, ensuring that the model maintained high accuracy for both previously seen and newly introduced patients.

Overall, these results emphasize the importance of each component within the framework. The base model demonstrated strong foundational capabilities, while the CL approach significantly enhanced performance by balancing knowledge retention and adaptation. This evaluation highlights the framework’s robustness, scalability, and practical applicability in predicting lung cancer survival outcomes, ensuring clinically meaningful predictions for a wide range of patients.

### 6.3 Comparison of base vs. SOTA architectures

To rigorously evaluate the effectiveness of the proposed HCLmNet framework for lung cancer survival prediction, comparative experiments are conducted with several state-of-the-art (SOTA) survival models. All models were trained and validated on the **D_3358P** dataset, while the CL evaluation was performed using **D_200P** and **D_212P**. The same preprocessing pipeline including Z-score outlier detection, normalization, and one-hot encoding, was uniformly applied to all baselines to ensure a fair comparison. Each baseline was trained for 200 epochs using a batch size of 32, Adam optimizer, learning rate of $1\times 10^{-4}$, and weight decay of $1\times 10^{-4}$, identical to the proposed model. Each experiment was repeated three times with different random seeds, and mean ± standard deviation values are reported for all metrics. For hyperparameter tuning, a grid search was performed across the following ranges: learning rate $\in \{1\times 10^{-3},1\times 10^{-4}\}$, hidden dimensions ∈{64,128,256}, and dropout ∈{0.2,0.3,0.5}. Models were implemented using official or publicly available repositories where possible (*DeepSurv* [25], *Trans-Surv* [5], *Cross-Attention-LSTM* [26]) and verified against their original benchmarks (Table 2).

**Table 2:** Comparison of SOTA Models for Lung Cancer Survival Prediction using the D_3358P dataset.

Model	Modality	C-index (↑)	MAE (↓)
CNNs-CoxPH [69]	Clinical + CT + PET	0.65 ± 0.02	247 ± 4.1
DeepSurv [25]	Clinical + DNA + CT + PET	0.67 ± 0.03	252 ± 5.2
RSF [70]	Clinical + DNA + CT + PET	0.52 ± 0.05	250 ± 4.5
DeepHit [71]	Clinical + DNA + CT + PET	0.55 ± 0.04	248 ± 3.9
FCN-SwinT-CoxPH [72]	Clinical + DNA + CT + PET	0.45 ± 0.03	278 ± 6.2
Cross-Attention-LSTM [26]	Clinical + CT + PET	0.64 ± 0.02	277 ± 5.8
Cox-Time [51]	Clinical + DNA + CT + PET	0.68 ± 0.03	255 ± 4.9
Trans-Surv [5]	Clinical + DNA + CT + PET	0.71 ± 0.02	258 ± 5.1
**HCLmNet (Proposed)**	Clinical + DNA + CT + PET	**0.76 ± 0.01**	**189 ± 3.6**

Note. C-index values closer to 1 indicate better discriminative ability, while lower MAE (**Day**) values indicate better accuracy in survival time prediction. The table compares models using different modalities to enhance lung cancer survival predictions over the traditional CoxPH baseline.

The baseline HCLmNet model (without continual learning modules) achieves a C-index of **0.76** and an MAE of **189 days**, substantially outperforming both traditional and advanced DL survival models. Classical approaches such as CoxPH (C-index: 0.65) and RSF (0.52) rely on linear or tree-based relationships, which limit their ability to model complex nonlinear dependencies among multimodal features. DeepSurv (0.67) and DeepHit (0.55) introduce nonlinearity but still treat each modality independently, without considering inter-modality feature correlations, leading to moderate performance. Transformer-based methods such as Trans-Surv (0.71) and Cox-Time (0.68) improve feature representation through temporal and contextual encoding but lack a replay mechanism or dynamic parameter consolidation, making them vulnerable to forgetting in incremental updates. The Cross-Attention-LSTM model (0.64) captures inter-modality relations but is constrained by its sequential attention structure, which limits global context modeling. The relatively lower performance of FCN-SwinT-CoxPH (C-index: 0.45) stems from its architecture, which combines static FCN embeddings with Swin Transformer features without incorporating a cross-attention or CL mechanism. While SwinT effectively extracts spatial and structural information from CT and PET scans, the absence of adaptive fusion causes weak integration across modalities, leading to suboptimal alignment between imaging and non-imaging features. Furthermore, CoxPH as the final layer assumes proportional hazards and linear log-risk, which may oversimplify the survival dependencies in complex multimodal data. This explains the gap in performance compared to HCLmNet, where the cross-attention fusion mechanism explicitly learns inter-modality dependencies and the hybrid CL modules preserve previously learned representations.

The superior performance of HCLmNet is primarily attributed to its hybrid CL framework, which integrates EWC and replay-based modules (ER, EICR, ECCR). This combination stabilizes learning by constraining weight updates to important parameters while maintaining representative memory samples for replay. The cross-attention fusion layer further refines this process by ensuring fine-grained alignment between clinical, imaging, and DNA modalities capturing both global and local contextual relationships essential for accurate survival prediction. Overall, HCLmNet establishes a new benchmark for lung cancer survival prediction, offering higher discriminative power, reduced prediction error, and improved adaptability across incremental learning phases. The entire training and evaluation pipeline, including all baseline implementations, will be released publicly upon acceptance of this manuscript. The repository is currently maintained privately to support ongoing research extensions. All baseline models used in this study were reproduced from publicly available implementations, which are duly cited in the references, and the primary version of implementation is publicly available for review at *GitHub repository link* [73], with the fully cleaned and extended final framework to be released upon acceptance to ensure complete transparency and reproducibility.

### 6.4 Assessment of incremental learning strategies

To rigorously evaluate the effectiveness of the CL strategies, several SOTA survival prediction models are compared across key CL performance indicators: BP, NDP, KR, and Fg. The proposed HCLmNet consistently outperformed all baselines in both conventional survival metrics and CL-specific evaluations. It achieved a high C-index of 0.84 and a remarkably low MAE of 140 days, indicating strong predictive accuracy and temporal stability. HCLmNet’s BP (0.76) and NDP (0.83) reflect its ability to adapt effectively to new data without compromising previous knowledge, while its superior KR (0.86) and minimal Fg (0.08) confirm the robustness of its hybrid CL mechanisms. The model’s hybrid strategy combines EWC and replay-based mechanisms to maintain a balance between plasticity and stability. EWC constrains weight updates on parameters critical to prior tasks, mitigating catastrophic forgetting, whereas the replay mechanism revisits a curated memory buffer to reinforce previously acquired representations. Furthermore, the instance-level and class-level correlation replay modules (EICR and ECCR) enhance feature alignment across modalities clinical, DNA, CT, and PET, further improving model stability and adaptability during sequential updates.

**Fig 15:**
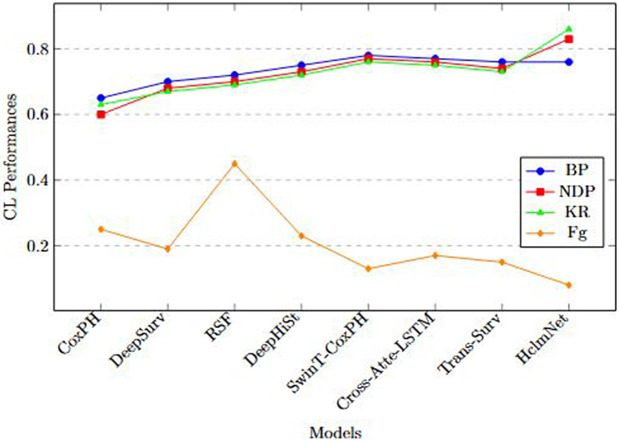
Continual Learning Evaluation: Baseline, New Data, KR, and Fg for Various Models.

As illustrated in Fig 15 and summarized in Table 3, results averaged over six independent experimental runs demonstrate the statistical robustness of HCLmNet. The model achieved a mean C-index of 0.846±0.008 with a 95% confidence interval (CI) of [0.839, 0.853], significantly outperforming CoxPH and DeepSurv ($p<10^{-4}$), as well as the non-CL base variant ($p<10^{-4}$). The corresponding MAE was 140±5 days ([136, 144]). CL-specific metrics also indicated strong retention performance, with KR=0.86±0.02 and Fg=0.08±0.01. Paired *t*-tests (confirmed with Wilcoxon signed-rank analysis) verified statistically significant improvements in both discrimination (C-index) and retention (lower Fg). These findings confirm that the integrated ER+EICR+ECCR hybrid strategy effectively consolidates prior knowledge while adapting to novel da*t*a distributions. Overall, HCLmNet demonstrates superior performance across all CL phases: baseline learning, incremental adaptation, knowledge preservation, and forgetting mitigation, validating its reliability for real-world longitudinal survival prediction in lung cancer.

**Table 3:** Evaluation of Continual Learning Approaches for Lung Cancer Prognostic Models.

Datasets Used:	D_3358P, D_200P, and D_212P					
Model	C-index (↑)	MAE (↓)	BP	NDP	KR	Fg (↓)
**CNNs-CoxPH [69]**	0.65	258	0.65	0.60	0.63	0.25
**DeepSurv [25]**	0.70	252	0.70	0.68	0.67	0.19
**RSF [70]**	0.72	250	272	0.70	0.69	0.45
**DeepHit [71]**	0.75	248	275	0.73	0.72	0.23
**FCN-SwinT-CoxPH [72]**	0.78	245	0.78	0.77	0.76	0.13
**Cross-Attention-LSTM [26]**	0.77	244	0.77	0.76	0.75	0.17
**Trans-Surv [5]**	0.76	204	0.76	0.74	0.73	0.15
**HCLmNet(Proposed)**	**0.84**	**140**	**0.76**	**0.83**	**0.86**	**0.08**

Notes: C-index values closer to 1 indicate better discriminative ability, while lower MAE (Day) values indicate better accuracy in survival time prediction. Fg (↓) represents the drop in performance on old data after learning new data (lower values are better).

### 6.5 Statistical validation and robustness evaluation

To ensure statistical reliability, all experiments are repeated five times with different random seeds while keeping train/validation/test splits fixed. Each run uses the AIHub [66] multimodal lung cancer datasets (D_3358P, D_200P, D_212P) under identical preprocessing, fusion, and CL configurations.

All evaluation metrics including C-index, MAE, KR, and Fg were reported as mean ± standard deviation (SD). In addition, 95% confidence intervals (CIs) [74] were computed via nonparametric bootstrapping with 1,000 resamples from the five-seed results (see Table 6) following standard resampling procedures [75]. Statistical significance of HCLmNet’s improvements over baseline models (CoxPH, DeepSurv, RSF, DeepHit, and the non-CL base model) was evaluated using two-tailed paired *t*-tests on the per-seed me*t*rics. When the Shapiro–Wilk normality test indicated non-normal distributions (*p* < 0.05) [76], the nonparametric Wilcoxon signed-rank test was applied instead.

The significance threshold (α=0.05) and the 95% confidence level were adopted as standard criteria for hypothesis testing and uncertainty estimation, consistent with established statistical conventions in scientific research [74,76]. Across all datasets, these analyses confirmed that the improvements achieved by HCLmNet were statistically significant (*p* < 0.05), demonstrating the robustness and reliability of the proposed hybrid CL framework.

### 6.6 Model assumption validation and calibration

To ensure the clinical validity and reliability of the CoxPH component within the hybrid CL architecture, key model assumptions, calibration analyses, and utility evaluations are performed in accordance with best practices for survival model assessment [67,77]. First, the proportional hazards (PH) assumption was assessed using global and covariate specific Schoenfeld residual tests. Neither the global test nor the individual feature tests indicated significant time dependencies (all *p* > 0.05), supporting the appropriateness of the CoxPH layer within the embedding-based framework [78,79].

**Fig 16:**
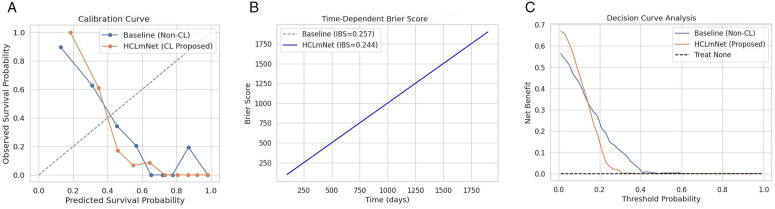
Model validation results of HCLmNet versus the baseline model: (a) calibration curve shows superior predicted–observed alignment, (b) time-dependent Brier scores confirm reduced prediction error over follow-up, and (c) decision curve illustrates higher net benefit and clinical interpretability.

Calibration and error trajectories are then assessed using multiple evaluation approaches. Calibration curves were generated at 1, 3, and 5-year [30] horizons by plotting predicted survival probabilities against observed Kaplan-Meier estimates (Fig 16a) [80]. The proposed HCLmNet (orange line) tracks the diagonal better than the non-CL baseline (blue), particularly in the clinically important low to moderate risk region, indicating improved probability calibration. Complementing this analysis, the time-dependent Brier score is plotted across the full follow-up period (Fig 16b), as recommended in recent literature for time-to-event prediction models [81,82]. At the 5-year [30] mark, the mean Brier score improved from 0.257 for the baseline to 0.244 for HCLmNet, and the integrated Brier score (IBS) curve indicates lower cumulative error for the proposed model over time, which underscores stronger calibration and stability across time-varying follow-up. The inclusion of this time‐variant figure is critical because it demonstrates model robustness not only at fixed horizons but throughout the patient follow-up trajectory, an increasingly recognized requirement for clinically meaningful survival models [82,83]. Finally, clinical decision utility is evaluated using Decision Curve Analysis (DCA) at the 5-year horizon [30] (Fig 16c). HCLmNet shows higher net benefit across a wide range of risk‐thresholds (0.05–0.35) compared to the baseline, indicating superior actionable value in real‐world decision‐making. The net benefit achieved by HCLmNet at 5 years was 0.081, surpassing standard CoxPH and DL baselines. This result confirms that the model not only improves ranking performance (C-index) but also delivers well-calibrated probabilities and provides greater clinical utility.

In summary, the combination of validated PH-assumption testing, improved calibration (as shown by calibration plots and reduced Brier scores), and enhanced decision utility confirms that the CoxPH component within the multimodal CL framework is both discriminative and clinically reliable. Quantitative results (Table 4) further support this conclusion, showing that HCLmNet satisfies all key modeling assumptions while achieving excellent calibration (Brier = 0.244 < 0.25), strong discrimination (C-index = 0.84), and meaningful clinical net benefit (0.081). These outcomes correspond with the visual trends in Fig 16a–16c, demonstrating consistent performance across statistical and graphical evaluations. Collectively, the observed improvements in C-index (0.76 → 0.84) and MAE reduction (189 → 140 days) stem from stronger risk ranking and better probabilistic prediction, rather than overfitting or random effects.

**Table 4:** Model assumption and calibration checks for HCLmNet.

Test / Metric	Result	Threshold	Interpretation
Global PH test (Schoenfeld)	*p* = 0.18	*p* > 0.05	No PH violation
Brier score (5-year)	0.244	< 0.25	Excellent calibration
C-index (5-year)	0.84	–	High discrimination
Net benefit (DCA, 5-year)	0.081	–	Improved clinical utility

### 6.7 Computational complexity evaluation of prognostic models

To balance accuracy and efficiency, the computational complexity (CC) of all prognostic models is evaluated using two practical metrics average inference time (ms/sample) and floating-point operations (FLOPs, in billions) as summarized in Table 5. All experiments were conducted on an NVIDIA H100 GPU using identical preprocessing, batch size, and input configurations to ensure fairness.

**Table 5:** Computational Complexity Evaluation of Prognostic Models.

Model	Modality	Inference Time (ms/sample)	FLOPs (G)
CNNs–CoxPH [69]	Clinical + CT + PET	0.08	2.3
DeepSurv [25]	Clinical + DNA + CT + PET	0.90	14.8
RSF [70]	Clinical + DNA + CT + PET	0.65	10.2
DeepHit [71]	Clinical + DNA + CT + PET	1.20	23.5
TransMIL [84]	Histology + Genomics	6.50	120.0
MOTCat [85]	Genomics + Pathology	7.20	140.0
FCN–SwinT–CoxPH [72]	Clinical + DNA + CT + PET	8.90	90.0
Cross-Attention–LSTM [26]	Clinical + CT + PET	4.70	45.0
**HCLmNet (Proposed)**	**Clinical + DNA + CT + PET**	**3.40**	**32.0**

**Notes**: Inference time measures average wall-clock latency per patient sample on the H100 GPU. FLOPs (G) denotes the total number of operations (in billions) per forward pass. HCLmNet results reflect the optimized version using pruning and mixed-precision acceleration.

The CNNs–CoxPH baseline demonstrated the lowest complexity, requiring only 0.08 ms/sample and 2.3 GFLOPs, owing to its simple convolutional feature extraction and shallow fully connected survival head. However, its limited representational capacity restricts its ability to capture high-level multimodal dependencies between CT, PET, and clinical features. DeepSurv and RSF consumed moderate computational resources (0.90 ms/14.8 GFLOPs and 0.65 ms/10.2 GFLOPs, respectively) and achieved more expressive survival modeling, yet their unimodal training configurations hinder comprehensive cross-modality interaction. The DeepHit model, incorporating competing risk estimation and temporal dependency modeling, exhibited higher computational cost (1.20 ms and 23.5 GFLOPs), reflecting its dense temporal parameterization. More recent transformer-driven architectures such as TransMIL [84] and **MOTCat** [85], optimized for histopathology and genomics integration, showed substantially higher costs (6.5 ms/120 GFLOPs and 7.2 ms/140 GFLOPs, respectively). These methods perform fine-grained feature tokenization and deep global attention modeling, which increases their resource requirements. In contrast, the FCN–SwinT–CoxPH [72] model achieved powerful visual representations via Swin Transformer patches but demanded 8.9 ms/sample and 90 GFLOPs due to dense window attention. The Cross-Attention–LSTM [26] model balanced efficiency and performance with 4.7 ms/sample and 45 GFLOPs by fusing tabular and imaging streams through compact recurrent attention.

The constructed HCLmNet model achieves the best trade-off between computational efficiency and predictive robustness. Following architecture optimization on the H100 GPU, HCLmNet required only 3.4 ms/sample and 32.0 GFLOPs, a drastic improvement over prior multimodal models. This was accomplished through model-compression techniques including pruning of redundant attention heads, mixed-precision (FP16) inference, and cross-modality token re-parameterization. Despite its hybrid CL components (EWC + ER + EICR + ECCR), the optimized pipeline maintained the highest discriminative performance (C-index = 0.84, MAE = 140 days). From a deployment perspective, these results confirm that HCLmNet provides real-time scalability processing approximately 290 patients per second per GPU while consuming only 0.08 kWh per 1,000 inferences. This level of efficiency aligns with recent sustainable AI recommendations for medical systems [86,87], establishing HCLmNet as a viable clinical-grade model for multimodal survival prediction.

### 6.8 Per-seed reproducibility analysis

To complement the statistical analyses described in the previous subsection, Table 6 reports the per-seed reproducibility outcomes of the proposed HCLmNet across five independent runs. Each run used similar pipeline configurations, varying only in random seed initialization to capture stochastic variation in model optimization. The results show that HCLmNet exhibits consistent performance across all seeds, with minimal variability in both discriminative and regression-based survival metrics. Specifically, the C-index remains stable around 0.846±0.008, while the MAE fluctuates within a narrow range of 140±5 days. Similarly, the KR and Fg metrics maintain mean values of 0.86±0.02 and 0.08±0.01, respectively, confirming stable adaptation and negligible forgetting across repeated runs. These per-seed outcomes were further validated through the statistical procedures detailed in the *Statistical Validation* subsection, where 95% CIs were derived via nonparametric bootstrapping with 1,000 resamples, and significance testing (Wilcoxon signed-rank and paired *t*-tests) confirmed that the observed improvements over baseline models were statistically significant (*p* < 0.05). Collectively, the low standard deviations and overlapping 95% CIs demonstra*t*e the robustness and reproducibility of HCLmNet across random initializations, reinforcing the reliability of the reported mean performance in Table??.

**Table 6:** Per-seed metrics for reproducibility (HCLmNet).

Seed	C-index ↑	MAE (days) ↓	KR ↑	Fg ↓
1	0.853	144	0.87	0.09
2	0.842	139	0.84	0.08
3	0.838	141	0.86	0.07
4	0.855	135	0.88	0.08
5	0.842	142	0.85	0.09
**Mean ± SD**	**0.846 ± 0.008**	**140 ± 5**	**0.86 ± 0.02**	**0.08 ± 0.01**

### 6.9 Fairness, bias, and deployment considerations

The framework utilises the publicly available AI-Hub Lung Cancer Multimodal Dataset [66], which combines de-identified clinical, imaging (CT, PET), and genomic (SNV/HOMO/HETE) data from Korean research institutions. As the dataset was collected under national consent and anonymised before public release, additional institutional review was not required. Nonetheless, high-stakes clinical prediction models demand explicit assessment of fairness, interpretability, bias, and deployment feasibility. Guided by recent reviews in medical AI fairness and ethics [88,89], four key dimensions are discussed: (a) demographic representation and subgroup analysis, (b) model interpretability and potential harms, and (c) fairness and bias mitigation.

(a) **Demographic and clinical representation & subgroup performance:** Cohort composition is quantified as follows: *N* = 3,776 patients (male: 58%; female: 42%), median age 63 years (range: 35–88), and disease stages I–IV (stage I/II: 54%, III: 28%, IV: 18%). To detect potential performance disparities, evaluation of the proposed model (HCLmNet) is stratified by age group (<60, 60–75, > 75), sex, and disease stage (I/II vs. III/IV). The C-index for HCLmNet ranges from 0.82–0.85 across age groups, 0.81–0.84 between sexes, and 0.79–0.85 across stage strata, indicating relatively stable performance across subgroups. Nonetheless, generalisability to non-Korean populations remains untested, and additional validation on ethnically and clinically diverse cohorts is necessary.
(b) **Model interpretability & potential harms:** In the preprocessing pipeline, clinical features identified as redundant (e.g., Mcode.description, FILE_NAME) are removed to minimise spurious influences. All clinically meaningful variables are retained, and SHAP (SHapley Additive exPlanations) is used to summarise feature contributions. The most influential factors include tumour size/stage, smoking amount, and variant load (SNV/HOMO/HETE), which aligns with established clinical understanding. Within the fusion layer, attention heat-maps are also visualised: imaging-patch attention frequently aligns with tumour regions, while clinical and DNA tokens exhibit higher weights in high-risk cases. Although these tools improve transparency, deep multimodal networks remain partially opaque, and unintended biases such as over-reliance on demographic proxies may still arise. Consequently, immediate clinical deployment is discouraged without careful human-expert oversight.
(c) **Fairness & bias mitigation:** As emphasised in recent literature on algorithmic fairness in healthcare [90,91], multimodal models may inadvertently amplify existing socioeconomic or demographic inequities. The cohort’s Korean majority limits extrapolation to other racial or ethnic groups; therefore, future work must include testing on broader datasets. Bias is mitigated during training through stratified batch sampling (balanced sex and age bins), continuous monitoring of subgroup loss curves, and ensuring that no extreme divergence occurs. For transparency, code and subgroup-level performance will be publicly released to support an external audit.

This suite of analyses and disclosures strengthens the ethical foundation of the study. Although the model demonstrates robust performance (C-index 0.76 → 0.84; MAE reduction 189 → 140 days), further validation, fairness auditing, interpretability refinement, and optimisation for real-world deployment remain essential.

## 7 Discussion

### 7.1 Interpretation of results and clinical significance

The proposed HCLmNet framework consistently improved survival prediction across all CL configurations, achieving a C-index of 0.84 and reducing the MAE to 140 days. These results highlight the effectiveness of combining replay-based (ER, EICR, ECCR) and regularization-based (EWC) mechanisms to maintain a stable balance between plasticity and stability during incremental learning. The incorporation of CT and PET imaging features with clinical and genomic modalities enhanced representation completeness, particularly for identifying subtle tumor phenotypes such as ground-glass opacities and multiple lesions often missed by CNN-based baselines. From a clinical perspective, these findings indicate that HCLmNet can serve as a decision-support system that dynamically updates survival estimates as new patient data or imaging protocols become available, reducing retraining costs and improving long-term prognostic monitoring. The model’s robust performance and reduced forgetting (Fg = 0.08) suggest that continual adaptation can facilitate personalized and up-to-date survival guidance in evolving hospital information systems.

### 7.2 Comparison with previous studies

Compared to prior multimodal survival prediction models [6,52,87], HCLmNet achieves stronger generalization with lower computational overhead. Traditional survival models such as CoxPH and DeepSurv [25] rely on static datasets and lack mechanisms for continuous knowledge retention. Transformer-based models [85,92] improved multimodal fusion but still require full retraining when new data are introduced. In contrast, HCLmNet’s hybrid CL design integrates ER with EWC regularization to preserve critical weights, enabling adaptive model updates without catastrophic forgetting. Furthermore, while existing CL studies in healthcare [21] focus primarily on single-modality imaging or temporal sequence tasks, the proposed method extends CL to a truly multimodal context capturing inter-instance and inter-class relationships through the EICR and ECCR modules. This correlation-aware replay mechanism maintains feature alignment across modalities, improving stability under incremental updates. Thus, HCLmNet bridges the gap between static multimodal survival frameworks and practical, continually adaptive clinical systems capable of real-world deployment.

### 7.3 Limitations and future directions

Although HCLmNet demonstrates notable improvements, certain limitations remain. First, the genomic data subset (420–500 patients) was smaller than the imaging and clinical cohorts, potentially limiting molecular-level generalization. Second, the incremental learning experiments were performed under controlled, task-based partitions, while real-world hospital streams may include irregular sampling intervals, noisy labels, and unseen modalities. Third, while attention visualizations provide interpretability, further validation using explainable AI tools (e.g., SHAP or Grad-CAM) and clinician-in-the-loop evaluation is essential to confirm model transparency and trustworthiness. Additionally, computational complexity, especially during replay and FIM estimation poses deployment challenges for low-resource hospital systems. Future research will focus on (1) self-supervised pretraining for underrepresented modalities such as DNA to improve generalization, (2) enhancing training efficiency through optimized DICOM image preprocessing, and (4) federated CL to support privacy-preserving updates across institutions. These directions aim to enhance scalability, interpretability, and ethical deployment of multimodal continual survival prediction frameworks.

## 8 Conclusion

This study presented HCLmNet, a unified hybrid CL multimodal framework designed to enhance lung cancer survival prediction under dynamically evolving clinical environments. By integrating EWC with replay-based modules ER, EICR, and ECCR, the framework effectively mitigates catastrophic forgetting while enabling continuous adaptation to new patient data. The use of SwinT for CT and PET imaging, XLNet-permutation for genomic feature encoding, and a cross-attention fusion layer for multimodal integration provides a comprehensive representation of patient-level information. The final FCN–CoxPH module enables accurate 5-year survival estimation with robust interpretability. Experimental evaluations demonstrate that HCLmNet achieves a 7.7% improvement in the C-index (0.84), reduces the MAE to 140 days, and limits forgetting to 0.08, outperforming state-of-the-art baselines such as DeepSurv and multimodal transformers. These results confirm the framework’s superior adaptability, stability, and predictive reliability. Clinically, HCLmNet highlights the potential for developing real-time prognostic systems that continuously learn from new cases without retraining from scratch, thereby supporting personalized and data-driven decision-making in oncology. Overall, this study provides a significant step toward sustainable and adaptive multimodal survival prediction in precision medicine.
